# U6 snRNA m6A modification is required for accurate and efficient splicing of *C. elegans* and human pre-mRNAs

**DOI:** 10.1093/nar/gkae447

**Published:** 2024-05-29

**Authors:** Aykut Shen, Katarzyna Hencel, Matthew T Parker, Robyn Scott, Roberta Skukan, Aduragbemi S Adesina, Carey L Metheringham, Eric A Miska, Yunsun Nam, Wilfried Haerty, Gordon G Simpson, Alper Akay

**Affiliations:** School of Biological Sciences, University of East Anglia, NR4 7TJ Norwich, UK; School of Biological Sciences, University of East Anglia, NR4 7TJ Norwich, UK; School of Life Sciences, University of Dundee, Dow Street, Dundee DD1 5EH, UK; Department of Biochemistry, University of Texas Southwestern Medical Center, Dallas, TX, USA; School of Biological Sciences, University of East Anglia, NR4 7TJ Norwich, UK; School of Biological Sciences, University of East Anglia, NR4 7TJ Norwich, UK; School of Life Sciences, University of Dundee, Dow Street, Dundee DD1 5EH, UK; Wellcome/CRUK Gurdon Institute, University of Cambridge, Tennis Court Rd, Cambridge CB2 1QN, UK; Department of Biochemistry, University of Texas Southwestern Medical Center, Dallas, TX, USA; Department of Biophysics, University of Texas Southwestern Medical Center, Dallas, TX, USA; Simmons Comprehensive Cancer Center, University of Texas Southwestern Medical Center, Dallas, TX, USA; School of Biological Sciences, University of East Anglia, NR4 7TJ Norwich, UK; Earlham Institute, Norwich Research Park, Norwich, UK; School of Life Sciences, University of Dundee, Dow Street, Dundee DD1 5EH, UK; Cell & Molecular Sciences, James Hutton Institute, Invergowrie, DD2 5DA, UK; School of Biological Sciences, University of East Anglia, NR4 7TJ Norwich, UK

## Abstract

pre-mRNA splicing is a critical feature of eukaryotic gene expression. Both *cis-* and trans-splicing rely on accurately recognising splice site sequences by spliceosomal U snRNAs and associated proteins. Spliceosomal snRNAs carry multiple RNA modifications with the potential to affect different stages of pre-mRNA splicing. Here, we show that the conserved U6 snRNA m6A methyltransferase METT-10 is required for accurate and efficient *cis-* and trans-splicing of *C. elegans* pre-mRNAs. The absence of METT-10 in *C. elegans* and METTL16 in humans primarily leads to alternative splicing at 5′ splice sites with an adenosine at +4 position. In addition, METT-10 is required for splicing of weak 3′ *cis-* and trans-splice sites. We identified a significant overlap between METT-10 and the conserved splicing factor SNRNP27K in regulating 5′ splice sites with +4A. Finally, we show that editing endogenous 5′ splice site +4A positions to +4U restores splicing to wild-type positions in a *mett-10* mutant background, supporting a direct role for U6 snRNA m6A modification in 5′ splice site recognition. We conclude that the U6 snRNA m6A modification is important for accurate and efficient pre-mRNA splicing.

## Graphical Abstract

**Graphical Abstract:**
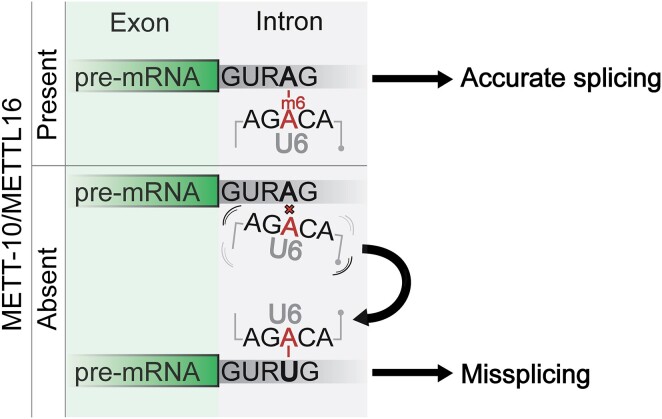

## Introduction

Recognition of 5′ and 3′ splice sites (SS) and the branchpoint sequence is crucial for the efficiency and accuracy of pre-mRNA splicing. How the spliceosome recognises the correct splice sites remains an important question in biology. In humans, 5′SSs are generally identified first by U1 snRNA through base pairing (1,2), which is followed by the U5 snRNA binding to the 5′SS exon sequences −3 to −1 (3,4), and U6 snRNA binding to the 5′SS intron sequences +3 to +5 (5). The 3′SS recognition is facilitated by the coordinated binding of the U2AF65–U2AF35 heterodimer to the 3′SS and the SF1/BBP and U2 snRNA to the branch point sequence (6,7). Following the recognition of 5′ and 3′SSs, U6 snRNA forms a helix with the U2 snRNA, leading to the formation of the B complex (8–11).

Splice site recognition is generally well-conserved among organisms with some differences. In *Caenorhabditis elegans* (*C. elegans*), only a small number of 5′SSs have the CAG//GUAAGU sequence that would form a continuous Watson-Crick-Franklin base pairing with the U1 snRNA as well as interacting strongly with the U5 and U6 snRNAs (12,13). Furthermore, *C. elegans* and likely other nematodes do not have a branchpoint consensus (14). Interestingly, *C. elegans* SF1/BBP retained the conserved branch point recognition domain, and the *C. elegans* U2 snRNA has the conserved sequence found in mammalian U2 snRNA that base pairs with the branch point motif (15). On the other hand, *C. elegans* introns have a relatively invariant UUUCAG/R 3′SS sequence (Supplementary Figure S1A). The short UUU sequence likely acts as the polypyrimidine tract recognised by the U2AF65, and U2AF35 recognises the CAG/R sequence for efficient splicing (16).

In addition to *cis-*splicing, nematodes, flatworms, cnidarians, rotifers, euglenozoa, and urochordates use spliced leader (SL) trans-splicing for mRNA maturation (17,18). SL trans-splicing replaces the 5′ ends of pre-mRNAs with the non-coding SL RNA (19). SL trans-splicing uses the same snRNAs U2, U4, U5 and U6, as in *cis-*splicing, except for U1 (20,21). In SL trans-splicing reactions, 5′ and 3′SSs are split between separate RNA molecules; the 5′SS resides within the SL RNA, and the 3′SS is generally positioned within the 5′ untranslated region of the pre-mRNA. Therefore, the trans-spliceosome needs to bring two separate RNA molecules together. The majority of *C. elegans* trans-spliced RNAs use SL1 non-coding RNA (80–90% of all trans-spliced RNAs), and the SL2 non-coding RNA is mainly used by the downstream genes in operons (7% of all trans-spliced RNAs) (22–24). The 5′SS on the SL1 RNA has an invariant AG//GUAAA, and most of the *C. elegans* 3′ trans-splice sites have the same UUUCAG/R sequence found in *cis-*spliced 3′SSs (Supplementary Figure S1A). The similarity of 5′ and 3′SSs between *cis-* and trans-splicing poses another challenge for the spliceosome during correct splice site recognition in *C. elegans*.

U5 and U6 snRNA binding preferences are important for 5′SS selection. In *Saccharomyces cerevisiae*, the U6 snRNA base pairs with the largely invariant 5′SS sequence and stabilises the B-complex formation (10). However, metazoan 5′SSs have degenerate sequences. Therefore, U5 and U6 snRNAs do not form continuous Watson-Crick-Franklin base pairing, and other factors play a role in stabilising U5 and U6 snRNA interactions with the pre-mRNA (5). Several modifications found on snRNAs contribute to snRNA-splice site interactions while recognising variable splice site sequences (25). For instance, Ψ28 of yeast U6 snRNA and the eight 2′O-methyl positions on human U6 snRNA all affect pre-mRNA splicing (26–28). In addition, U6 snRNA m2G72 modification plays an important role during pre-mRNA splicing in human cells (29,30). Furthermore, U6 snRNA is m6A modified in many organisms by the conserved RNA methyltransferases METTL16 (human)/FIO1 (*Arabidopsis thaliana*)/METT-10 (*C. elegans*)/MTL16 (*Schizosaccharomyces pombe*) (31–36). Mutations in the *S. pombe* MTL16 and *A. thaliana* FIO1 lead to the mis-splicing of 5′SSs with adenosine at +4 position (+4A), and the alternative 5′SSs tend to have +4U together with a stronger U5 binding motif (33,36). However, *in vivo*, direct evidence supporting a functional interaction between the U6 snRNA m6A and 5′SS +4A is missing.

In addition to U6 snRNA methylation, mammalian METTL16 can methylate the pre-mRNA of S-adenosylmethionine (SAM) synthetase MAT2A at a UACAGA motif that mimics U6 snRNA structure (31,32,37–40). METTL16 and its catalytic activity are essential for mouse embryonic development (35,40). Although METTL16’s role in U6 snRNA and MAT2A methylation is well established in humans and mice, its role in pre-mRNA splicing regulation has not been extensively studied. Recent studies have also implicated METT-10 in mRNA m6A modification and SAM homeostasis in *C. elegans*. METT-10 has been reported to methylate the pre-mRNAs of three SAM synthetase genes, *sams-3, -4* and *-5*, at the -2A position of the 3′SS UACAG//A sequence leading to intron retention and alternative 3′SS usage (35,41). However, these studies failed to identify global pre-mRNA splicing defects in *mett-10* mutant animals (35,41).

In *C. elegans*, METT-10 is required for germ cell proliferation and larval development (42). Without METT-10 function, germ cells arrest during mitosis and fail to enter meiosis. *mett-10* mutant animals develop multiple larval developmental defects and have embryonic lethality. The strong phenotypes associated with the loss of METT-10 function aren’t fully accounted for by its mRNA targets (35), suggesting that U6 snRNA m6A modification by METT-10 could be essential for organismal development.

To understand the transcriptome-wide functions of METT-10 in *C. elegans*, we used short- and long-read sequencing to comprehensively analyse the *cis-* and trans-splicing of pre-mRNAs alongside their expression. Our results show that METT-10 loss-of-function causes global splicing defects in *C. elegans*, with both the *cis-* and the trans-spliceosomes failing to splice hundreds of genes accurately or efficiently. We identify the 5′SS +4A as a critical determinant of *cis-*splicing in the absence of U6 snRNA m6A modification in *C. elegans* and human cells. We further show that strong U5 and U6 interactions at the 5′SSs can support splicing at weak 3′SS sequences. As the SL RNA 5′SS is invariant, we determine that most trans-splicing defects result from weak 3′SSs based on U2AF binding motifs. We find the spliceosomal protein SNRP-27/SNRNP27K functions with the m6A-modified U6 snRNA for accurate splicing of 5′SSs. Finally, we provide the first *in vivo* evidence for a direct role of U6 snRNA m6A modification in recognising 5′SS +4A by editing a 5′SS +4A to +4U and thereby restoring accurate splicing in *C. elegans* pre-mRNAs in the absence of U6 snRNA m6A modification.

## Materials and methods

### Nematode culture, strains and maintenance

*C. elegans* strains were grown on Nematode Growth Medium (NGM) agar plates with *Escherichia coli* HB101 strain as a food source and maintained at 20°C unless stated otherwise. The following strains were used in the experiments: wild-type N2 Bristol, ALP010 *mett-10(ok2204*) III derived from backcrossing of VC1743 (42), ALP012 p*Mex-5*::*mett-10*::OLLAS::*tbb-2_UTR* (rna004) II generated using MosSCI (43), ALP113 (*mett-10(ok2204)III; pipp-4P(rna088* [*pipp-4P* intron 4 +4A > U]) generated by CRISPR/Cas9 genome editing. All experiments were performed starting from synchronous L1 animals, generated by hypochlorite treatment in a 2:2:1 solution (sodium hypochlorite (4–5%), H_2_O and 10 M NaOH).

### Worm collection and RNA extraction

*C. elegans* strains N2 Bristol, ALP010, and ALP012 were grown on *E. coli* HB101 and collected at the young adult stage. RNA was isolated using TRIsure (Bioline, Cat. No. BIO-38032) following standard phenol-chloroform RNA extraction. The purity of the RNA was checked with Nanodrop, and the integrity was quantified using the Agilent 2200 TapeStation System. RNA concentration was measured with Qubit using RNA HS Assay (Invitrogen™ Q33224).

### Oxford Nanopore Technologies direct RNA sequencing

100 μg of total RNA was used to perform poly(A)+ RNA isolation using PolyATtract® mRNA Isolation Systems (Promega UK LTD, Cat. No. Z5310). 750 ng of recovered mRNA was used for library preparation using the direct RNA sequencing (DRS) kit following the manufacturer's instructions (Oxford Nanopore Technologies, SQK-RNA002). Libraries were quantified using Qubit dsDNA HS Assay (Invitrogen™ Q32851). Sequencing was done in-house on Oxford Nanopore Technologies (ONT) MinION devices using flowcells R9.4.1.

### Illumina RNA sequencing

1 μg of total RNA was used to perform mRNA isolation using NEBNext Poly(A) mRNA magnetic isolation module (NEB Cat. No. E7490). The resulting poly(A)+ RNA material was used to prepare the libraries using the NEBNext® Ultra II Directional RNA Library Prep Kit for Illumina® (NEB, Cat No. E7760S) following the manufacturer's instructions. Libraries were quantified using Qubit dsDNA HS Assay (Invitrogen™ Q32851). Paired-end 150 bp sequencing was done at the Novogene (UK) Company Limited using an Illumina NovaSeq 6000.

### Egg-laying assay

Synchronous L1 animals were plated on *E. coli* HB101-seeded NGM plates at 20°C. Animals were grown to the larval stage L4 and shifted to 15, 20 and 25°C. Each animal was transferred to a new plate twice daily. Egg-laying was performed with one technical replicate per biological replicate (genotype); four animals for N2 and ALP012 and eight for ALP010 were used. Eggs were counted for 3 days using biological triplicates. One-way ANOVA was used to test significance in the egg-laying assay, followed by a Tukey's post-hoc test. Normality and homogeneity of variances were, respectively, assessed by Shapiro-Wilk and Leven tests. The figure was generated using SuperPlots (44).

### Developmental assay

Synchronous L1 stage animals were individually picked onto *E. coli* HB101 seeded NGM plates and exposed to different temperatures. The developmental stages of animals were determined in biological triplicate using a stereomicroscope 42 hours post L1 and classified as L2-L3, late L4 and young adult.

### ONT direct RNA sequencing data processing

Read signals were base called using Guppy version 5.0.11 in GPU mode, employing the high-accuracy RNA model with the parameters -x cuda:all:100%, –num_callers 24, –calib_detect, –reverse_sequence yes, and -c rna_r9.4.1_70bps_hac.cfg. For mRNA modification analysis with *Yanocomp* (45), reads were aligned to the WBcel235 *Caenorhabditis* reference transcriptome (Ensembl release 95) and the *de novo* assembled transcriptome (described below) using minimap2 version 2.17 (46) with the following parameters: -a -L –cs = short k14 –for-only –secondary = no. For other analyses, reads were aligned to the WBcel235 *Caenorhabditis* reference genome using a two-pass alignment approach with minimap2 (46) and 2passtools version 0.3 (47). For transcriptome assembly and differential error site analysis, the first pass alignments were generated with the following parameters: –splice, -k 14, -uf, -w 5, -g 2000, -G 200000, –end-seed-pen 15, -A1, -B2, -O2,32, -E1,0, -C9, –splice-flank = yes, and -z200. High-confidence splice junctions from each replicate were extracted using 2passtools score and then merged using 2passtools merge to create a final set of trusted splice junctions. In the second pass, the reads were realigned to the WBcel235 reference genome using minimap2 with the same parameters as before, in addition to using the trusted junctions as a guide with the parameters -junc-bed and -juncbonus = 10. For outron retention analysis, the first pass alignments were generated using minimap2 with parameters: -ax splice, -uf, -L, –cs, -k 14, and -G 50370. High-confidence splice junctions were extracted using the 2passtools score and merged into a final set of trusted splice junctions using 2passtools merge. Second-pass alignments were generated using the following parameters: -ax splice, -uf, -L, –cs, -k 10, and -G 50370, along with the trusted junctions from the first-pass ONT-DRS alignments.

### ONT-DRS poly(A)+ mRNA modification analysis

Two computational methods were used to identify METT-10 dependent RNA modifications: First, differential modification analysis was conducted at the signal level using the ‘n-sample’ GitHub branch of *Yanocomp* (45) by aligning the reads to the WBcel235 *C. elegans* reference transcriptome or the *de novo* assembled transcriptome, followed by generation of kmer-level signal data using f5c event align version 0.7 (48,49) and *Yanocomp* prep. A three-way comparison of the genotypes was performed using the *Yanocomp* gmmtest, with a minimum KS statistic of 0.25. A false discovery rate (FDR) of 0.05 was applied as a cutoff to identify transcriptomic sites with significant changes in the modification rate. For the second method, differential error site analysis was conducted using ‘*differ*’ (50) by aligning the reads to the genomic sequence using 2-pass alignment (as described above) and pairwise comparisons were conducted for different combinations of genotypes (wild-type, ALP010, and ALP012) with a median expression threshold of 5 and with or without CPM normalisation. An FDR of 0.05 was applied as a cutoff to identify genomic sites with significant changes in the error rate.

### Identification of trans-spliced sites

Two different methods were employed to identify trans-spliced sites: (i) detection from ONT direct RNA sequencing data through pairwise alignment of 5′ ends to the SL leader sequence and (ii) detection from Illumina RNA-seq data through SL leader sequence trimming (51). For the first method, wild-type, ALP010 and ALP012 direct RNA sequencing data were used to predict nearby acceptor sites by searching for an ‘AG’ motif that could serve as a trans-splicing site within 10 nt of the aligned 5′ end of reads. The known sequence of splice leaders (SLs) and the 24 nt genomic sequences downstream of the predicted acceptor site of the exon were concatenated to create an expected reference sequence for the trans-spliced mRNA. Smith-Waterman local alignment of this reference sequence against the 5′ ends of the sequence of the ONT-DRS read (24 nt downstream of the aligned 5′ end plus up to 28 nt of the 5′ soft-clipped portion of the read) was then performed with the parasail alignment function sw_trace_striped_32 (52). For each read, the trans-splicing position and splice leader class (SL1 or SL2) with the best pairwise alignment score were retained. The quality of the pairwise alignment across the trans-splice site was assessed using the junction alignment distance, which is defined as the minimum distance from the splice junction to the first alignment error (47). Trans-splice sites where the junction alignment distance of the highest scoring read was >8 nt were retained as high-scoring putative trans-splice sites. These trans-splice sites were used to create a position-specific scoring matrix (PSSM) for the outron motif, which was then used to score all possible trans-splicing sites—a threshold for the PSSM score was determined by splitting the scores of all possible sites at deciles and identifying the decile that maximized the Chi^2^ statistic of the thresholded junction alignment distances. Finally, a high-confidence set of trans-splicing sites was generated by retaining positions with both a maximum junction alignment distance of 8 and a position-specific matrix score above the threshold. For the second method, SL-like sequences were trimmed from the 5′ end of the forward reads of Illumina data using cutadapt with a minimum overlap of 7 nt, a maximum error rate of 0.09, and a minimum length (after trimming) of 15 nt (51). For each read, the trimmed SL sequence and class (SL1 or SL2) were recorded. The reads were then aligned to the WBcel235 reference genome using STAR, as described below. Positions with more than 10 aligned reads with a trimmed SL sequence were identified as candidates. False positives were filtered out by identifying the candidate sites where the upstream genomic sequence was identical to the trimmed SL sequence. High confidence sites were used to build a PSSM for the outron sequence and select a PSSM threshold, as described above. Sites with a minimum of 2 supporting reads and an outron sequence scoring above the threshold were retained as high-confidence trans-splicing sites. The sites from the Illumina and ONT-DRS methods were combined to generate a list of non-redundant trans-splice sites.

### Detection of METT-10 dependent trans-splicing defects

METT-10-dependent trans-splicing defects were detected by developing scripts to assess changes in trans-splicing levels per annotated trans-splice site between wild-type, ALP010 and ALP012 genotypes pairwise. The annotated trans-splice site positions were assigned to gene features in Ensembl release 95 reference annotation using pybedtools version 0.9.0 (53,54). In cases where sites overlapped with multiple genes, they were assigned to the genes whose 5′ exon boundaries showed more significant agreement with the annotated trans-splice site positions. In cases of a tie, trans-splice sites were assigned to both genes being compared. Overlapping reads with each annotated trans-splice position were identified using pysam version 0.21.0 (55). Reads were categorised as ‘trans-spliced’ or ‘retained outron’ based on whether the 5′ position of the read end fell within the −14 to + 10 window of the trans-splice site or earlier (<−14), respectively. Reads categorized as ‘retained outron’ were conditioned to have identical splice junctions immediately downstream of the trans-splice site as those categorised as ‘trans-spliced’. The relative proportions of each category were counted. Counts from replicates of the same genotype were aggregated, and a 2 × 2 contingency table was created for each annotated trans-splice site, comparing the two genotypes. Significantly altered trans-splicing profiles were identified by performing a likelihood-ratio test (G-test) using scipy (56). For sites with a *P*-value of <0.05, G-tests for homogeneity between replicates of the same genotype were conducted. Calculated *P*-values were adjusted for multiple testing using the Benjamini–Hochberg false discovery rate (FDR) method. Retained outrons that were also *cis-*spliced at the annotated trans-splice positions and did not have an annotated SL site on the upstream exon with the 5′ splice site were classified as ‘*cis-*spliced retained outron’ (CSRO). Retained outrons that exhibited a higher difference in the proportion of trans-spliced (PSI) reads at a downstream SL site on the same exon were classified as ‘Alternative 3′ trans-splice site’. The remaining trans-splice sites that showed a significant difference in PSI were classified as ‘retained outron’ (RO). Sequence logos per alternative splicing event were generated using matplotlib version 3.7.1 (57) and matplotlib_logo (https://github.com/mparker2/matplotlib_logo). Significant differences between the motifs were identified using a G-test. Contingency tables of splice site classes at U2AF65 and U2AF35 interacting positions were generated by analysing the difference in positions (−6 to −4) of the 3′ trans-splice sites from the consensus motif UUU and the difference in positions (−3 to + 1) of the 3′ trans-splice sites from the consensus motif CAGR, respectively. Heatmaps of contingency tables were generated using Seaborn version 0.12.2 (58). Gene tracks, utilising reads aligned to the WBCel235 reference genome, were generated using pyBigWig version 0.3.18 (59), pysam version 0.21.0 (55), and matplotlib version 3.7.1 (57)

## Illumina RNA sequencing data processing

### Genome alignment

Standard adapter sequences and common contaminants were removed from the paired-end reads using BBduk from the BBtools package version 37.62 (https://sourceforge.net/projects/bbmap/) with the following parameters: -k 21, -ktrim r, -hdist 1, -mink 11, -trimq 15, -qtrim rl, -minlen 35, -tpe, and -tbo. The quality of the reads was assessed using FastQC version v0.12.1 (https://www.bioinformatics.babraham.ac.uk/projects/fastqc/) and MultiQC version 1.13 (60). The reads were aligned to the WBcel235 *C. elegans* reference genome using STAR version 2.7.10b (61) with the following parameters: –outFilterMultimapNmax 3, –alignSJoverhangMin 8, –alignSJDBoverhangMin 3, –outFilterMismatchNmax 4, –alignIntronMin 39, –alignIntronMax 20000, –chimOutType Junctions, –chimSegmentMin 15, –chimScoreJunctionNonGTAG 0, and –chimSegmentReadGapMax 20000. A splice junction database was generated from the Ensembl release 95 reference annotation with the parameters –genomeSAindexNbases 12 and –sjdbOverhang 149.

### Transcriptome assembly

Condition-specific transcriptome assemblies were generated using Stringtie version 2.1.7 (62) from the pooled Illumina and complementary ONT-DRS alignments using the following parameters: –mix, –rf, -c 1, -s 1, -g 0, and -M 10. A unified set of transcripts was created by merging all resulting condition-specific assemblies with the Ensembl release 95 reference annotation using the Stringtie merge tool with parameters: -g 0, -F 0, -T 0, -f 0.001 and -i. Open reading frames were annotated using Transuite (63) version 0.2.2 with parameters: –cds 50 and –ptc 90, which were used in outron retention analysis to assign SL sites to genes they likely originate.

### Transcript quantification and alternative splicing

Transcript quantification and splicing analysis was done as previously described in Parker *et al.* (33). Assembled transcripts were quantified per Illumina RNA-seq sample using Salmon version 1.10.1 (64) with the parameters -l A and –validateMappings. The WBcel235 *C. elegans* reference genome assembly was used as a decoy. Local splicing events in the assembled annotation file were classified using SUPPA version 2.3 (65) with the parameters generateEvents, -f ioe, –pool-genes, and -e SE SS MX RI FL. Event-level relative abundance (PSI) values per sample for each local event were then estimated from the transcript-level quantifications with the parameters psiPerEvent and –total-filter 1. PSI values combined from all samples were loaded into Python version 3.8.12 using pandas version 1.0.1 (66–68). The relationship between genotype and PSI was tested by fitting generalised linear models (GLMs) per local splicing event using statsmodels version 0.11 (69). Calculated p-values were adjusted for multiple testing using the Benjamini-Hochberg false discovery rate (FDR) method. Local splicing events with significant changes in PSI between ALP010 and wild-type strains were identified using an FDR threshold of 0.05. Sequence logos per alternative splicing event were generated using matplotlib version 3.7.1 (57) and matplotlib_logo. Significant differences between the motifs were identified using a G-test. Contingency tables of splice site classes at U5 and U6 interacting positions were generated using the difference of the −2 to −1 positions of the 5′SS from the consensus motif AG and the difference of the + 3 to + 5 positions of the 5′SS from the consensus motif RAG, respectively. Heatmaps of contingency tables were generated with Seaborn version 0.12.2 (58). Gene tracks using reads aligned to the WBCel235 reference genome were generated using pyBigWig version 0.3.18 (59), pysam version 0.21.0 (https://github.com/pysam-dev elopers/pysam) and matplotlib version 3.7.1 (57).

The effect size for each position around the 5′SS was calculated by taking the difference between the distribution of ΔPSI for splice sites which had a particular base at the position and the distribution of ΔPSI for splice sites which did not have the base. The significance was tested with a Wilcoxon signed-rank test. Where the resulting *P*-value was <0.01, the presence of a base at a particular site was determined to have a significant effect on the likelihood of an alternate splicing event occurring at the 5′SS splice site. The direction of the effect was calculated by comparing the proportion of splice sites with a given base at a given position between *mett-10^−/−^* and wild-type to the proportion of splice sites without the given base that had a significant difference in alternate splicing. Where the proportion of events was higher in sites with the base, sites with that base at a given position were less favoured in the mutant and were given a negative sign. The signed significance of each base was plotted against the position in the 5′SS, with points sized according to the frequency at which the base occurred at a given position across all 5′SS in the genome.

### Differential gene expression analysis

Gene expression analysis was conducted using the Illumina RNA sequencing data. The abundance of transcripts was estimated through pseudoalignment with Salmon version 0.11.2 (64). Aggregation of counts to the gene level was achieved using tximport (70). Differential gene expression analysis, comparing the *mett-10* mutant with the wild-type, was performed in R version 3.5, utilising edgeR version 3.22.5 (71). Genes exhibiting differences in their relative expression were identified using an FDR cutoff of 0.05. The resulting list of genes was then used to examine the relationship between the alternative splicing and the gene expression.

### Identification of upstream start codons in outron regions

The outron start position was estimated for RO events from the ONT-DRS reads contributing to the RO class. The outron start position for CSRO events was estimated from the transcripts assembled by Stringtie2, sharing the identical 3′SS position with the trans-splice site. Sequences from exonic regions extending from the outron start to the canonical start codon were extracted and analysed to determine the existence of additional start codons. A few RO and CSRO events presented multiple isoforms with unique exonic regions. This was caused by alternative splicing occurring upstream of the canonical start codon, altering the sequence context within the outron region. In this case, the analysis was conducted individually for each isoform.

### Kozak motif analysis

To select a single candidate per retained outron, we utilised all start codons annotated in the WBcel235 (ver. 95) reference to create a position-specific scoring matrix (PSSM) for the positions −3 to −1 relative to the ATG codon. The log-likelihood scores of Kozak sequences for each in-frame start codon were then calculated using the PSSM. Start codons with the highest scores were selected for further analysis and plotting.

### Processing and alternative splicing analysis of public Illumina RNA-seq datasets

The SNRP-27 Illumina RNA-seq dataset was obtained from the GSA under accession GSE113275 (72). The data was processed and analysed similarly to that described for the METT-10 data. However, instead of assembling a new transcriptome for each condition, we used pre-assembled transcripts from the METT-10 and N2 datasets. These transcripts were then quantified using Salmon for the strains CB936 *unc-73*(e936)I and SZ118 *unc-73*(e936)I; *snrp-27*(az26)I. For subsequent analyses, we followed the same methods as described above. Pearson's chi-square test of independence was applied to assess the likelihood of concurrent usage of splice sites in *mett-10^−/−^* and SNRP-27 M141T. Significant differences between the motifs were identified using a G-test.

The Illumina RNA-seq datasets for METTL16 and THUMPD2 were obtained from GEO accessions GSE90914 (31) and GSE219260 (29), respectively. The data processing and analysis were performed similarly to the METT-10 data but with a few differences. Reads were aligned to the human reference genome GRCh38 with a splice junction database generated from the Ensembl GRCH38 release 109 annotations. The METTL16 dataset had longer reads (∼125 bp) compared to the THUMPD2 dataset (∼51bp). In addition, we were interested in determining whether splicing changes dependent on METTL16 could also be observed in THUMPD2 KO. Therefore, the pooled alignments of METTL16 KD and control 293A-TOA cell lines were used for generating condition-specific transcriptome assemblies. A unified set of transcripts was created by merging all resulting condition-specific assemblies with the Ensembl GRCh38 release 109 annotations. Subsequent transcript quantification and splicing analysis for both datasets were performed using the assembled transcriptome as described above. Significant differences between the motifs were identified using a G-test.

### Sequencing data

All RNA sequencing raw data have been deposited to the European Nucleotide Archive with the accession number PRJEB65287. The RNA-Seq data used for the SNRP-27 is described in Zahler *et al.* ((72). The RNA-Seq data for METTL16 knock-down cell lines is described in (31), and the RNA-Seq data for THUMPD2 knock-out cells is described in (29).

### Recombinant METT-10 protein purification

Full-length codon-optimised *C. elegans* METT-10 (Supplementary Figure S1B) was subcloned into the pET-21a (Novagen) vector between NdeI and XhoI sites for expression in *E. coli*. The clone was verified by Sanger sequencing. The construct was overexpressed in Rosetta (DE3) cells (Novagen) using autoinduction media (73). Soluble METT-10 was purified from a soluble lysate using Nickel affinity chromatography (Ni-NTA) and further purified by ion-exchange chromatography and gel filtration chromatography.

### *In vitro* methylation assay

The *in vitro* methylation assay was carried out as described by Wang *et al.* (74). Briefly, a 15 μl reaction mixture containing 50 mM Tris pH 8.5, 0.01% Triton X, 50 μM ZnCl_2_, 1 mM DTT, 0.2 U/μl RNasin, 1% glycerol, 1 μM [3H]-SAM (Perkin Elmer), 1 μM full-length *C. elegans* U6, and 750nM METT-10 was incubated at room temperature for 1 hr. The reaction mixture was blotted on Biodyne B nylon membranes and washed with buffer (20 mM Tris pH 7.5, 0.01% Triton X), deionized water, and 95% ethanol, in that order, and then subjected to liquid-scintillation counting using the TriCarb 2010 TR Scintillation Counter (Perkin Elmer). RNA levels with the incorporated 3H-methyl group are shown as disintegrations per minute (DPM). All *in vitro* methylation data are shown as mean ± SD from three replicates. The RNA substrate, *C. elegans* U6 RNA, was transcribed *in vitro* and purified after separating on a denaturing polyacrylamide gel (75). The template sequence encoding the *C. elegans* U6 is 5′- GTTCTTCCGAGAACATATACTAAA ATTGGAACAATACAGAGAAGATTAGCATGGCCCCTGCGCAAGGATGACACGCAAATTCGTGAAGCGTTCCAAATTTTT -3′.

### CRISPR/Cas9 genome editing

For the generation of the strain ALP113 (*mett-10(ok2204)III; pipp-4P(rna088* [*pipp-4P* intron 4 +4A > U]), we used custom-designed IDT Alt-R™ crRNA 5′-ACCCACTCAACTCCTTACAC-3′ together with pre-made IDT Alt-R™ tracrRNA and Alt-R™ S.p. Cas9 Nuclease V3. Injection mixes were prepared as described in (76). We used IDT Alt-R™ modified homology-directed repair template 5′-CGGATGTGCTCTTTATACAGCTTACAAATCATTCAATTATTATACGTGCAGGAAATCGGAAATTATTGGGAAAGTGTAtGGAGTTGAGTGGGTTCGAGTTGAAAATTCAGTGGAAATTATGAAAAGCGATCGCTTCAATGGAGAAATC-3′.

### RT-PCR and restriction digestion analysis

RNA was extracted from synchronized young adult animals using Trisure and chloroform extraction for cDNA synthesis, as described above. cDNA was synthesized from 2μg of total RNA using Bio-Rad iScript Reverse Transcription Supermix (1708840) following manufacturer recommendations alongside no-RT control. 2 μl of the cDNA was used as a template in PCR reactions with forward primer 5′-TGGTGCCGATTCTGATTGGGCT-3′ and reverse primer 5′-TTGGATTTCAGCCGGGTGAATG-3′ matching to *pipp-4P* (WBGene00022167) exon-4–exon-5 junction. Restriction digestion was carried out using the NEB enzyme Hpy166II (R0616). Samples were analysed using a 2% agarose gel in TCA or 3% agarose in a TBE buffer. Image analysis was done using FIJI (77).

## Results

### Loss of METT-10 function leads to *cis-* and trans-splicing defects

*mett-10(ok2204) null* mutants (*mett-10^−/−^* hereafter) have a deletion removing the entire methyltransferase domain (42) and have been shown previously to lack detectable U6 snRNA m6A modification (35). Consistent with this, we found that a recombinant METT-10 protein can methylate *in vitro* transcribed U6 snRNA (Supplementary Figure S1B). To examine the dependency of the developmental phenotypes on *mett-10* deletion, we complemented the *mett-10^−/−^* with a single-copy insertion transgene expressing METT-10 protein under the control of a germline promoter (78) in the *mett-10^−/−^* background (*mett-10 rescue*). As expected, germline expression of *mett-10* significantly rescued the fertility defects in *mett-10^−/−^* animals at all temperatures tested (Supplementary Figure S1C). Notably, germline expression of *mett-10* in *mett-10^−/−^* animals also rescued somatic developmental defects, and animals appeared like wild-type (Supplementary Figure S1D).

**Figure 1.**
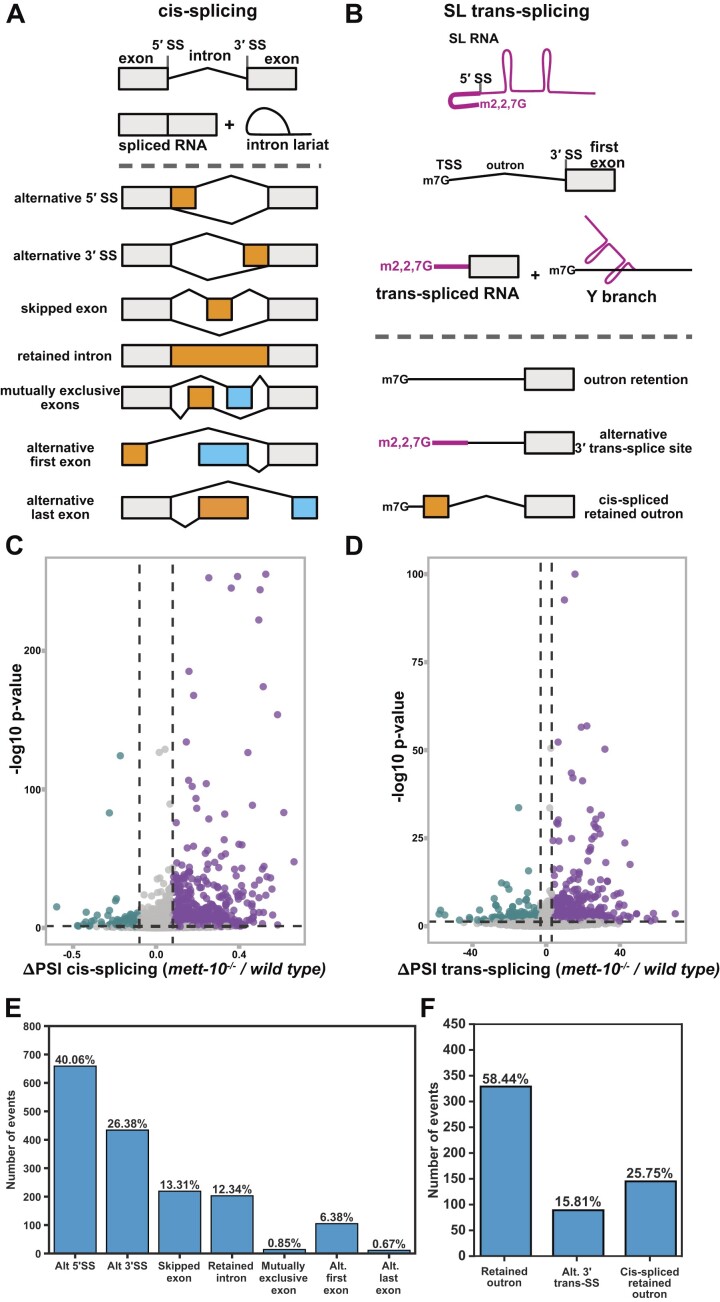
The absence of *mett-10* causes *cis*- and *trans*-splicing defects. (**A**) Overview of pre-mRNA *cis*-splicing and *cis*-splicing defects detectable by RNA sequencing. The grey bars represent exons adjacent to alternative splicing events. Orange and blue bars represent sequences that are excluded or included depending on the alternative splicing event type. (**B**) Overview of pre-mRNA trans-splicing and trans-splicing defects detectable by RNA sequencing. (**C**) Volcano plot of all transcripts tested for *cis*-splicing changes between *mett-10^−/−^* and *wild-type* animals. The X-axis shows PSI differences between *mett-10* and wild-type animals, and the y-axis shows the significance in *P*-values. Transcripts that show increased splicing in *mett-10^−/−^* (p ≤ 0.05 and ΔPSI ≤ −0.1) at a given splice site are coloured green, and transcripts that show reduced splicing in *mett-10^−/−^* (*P* ≤ 0.05 and ΔPSI ≥ 0.1) at a given splice site are coloured purple. (**D**) Volcano plot of all transcripts tested for trans-splicing changes between *mett-10^−/−^* and *wild-type* animals. Transcripts that show increased splicing in *mett-10^−/−^* (*P* ≤ 0.05 and ΔPSI ≤ −3) at a given splice site are coloured green, and transcripts that show reduced splicing in *mett-10^−/−^* (*P* ≤ 0.05 and ΔPSI ≥ 3) at a given splice site are coloured purple. −log_10_*P*-value of 1 gene has been lowered to fit into the graph. (**E**) Classification of all significant (FDR < 0.05) *cis*-splicing defects in *mett-10^−/−^* animals. (**F**) Classification of all significant (*P*-value < 0.05) trans-splicing defects in *mett-10^−/−^* animals.

We took a global RNA-sequencing approach to understand the impact of the loss of METT-10 function on gene expression. We used Oxford Nanopore Technologies Direct RNA Sequencing (ONT-DRS) of poly(A)+ RNA purified from wild-type, *mett-10^−/−^* and *mett-10 rescue* animals and Illumina paired-end 150 bp sequencing of poly(A)+ RNA purified from *mett-10^−/−^* and wild-type animals to analyse isoform-specific gene expression and alternative splicing (Figure 1A and B). Short-read sequencing generates relatively high-coverage data with greater statistical power to quantify *cis-*splicing. The ONT-DRS generates long-read data extending from the polyA-tail to the 5′ ends of transcripts, allowing the detection of individual RNAs that are either trans-spliced or not. We used three biological replicates per genotype for the ONT-DRS and four biological replicates per genotype for the Illumina sequencing. We obtained, on average, 2.8 million reads per replicate in ONT-DRS with an average mapping efficiency of 99% and 50 million reads per replicate in Illumina sequencing with an average mapping efficiency of 98% (Supplementary Table S1). We quantified the *cis-*splicing defects using Illumina reads (Figure 1A and C), and the trans-splicing defects using ONT-DRS reads (Figure 1B and D). We mapped reads to a custom *C. elegans* reference transcriptome built using wild-type and *mett-10^−/−^* Illumina and ONT-DRS reads to capture novel transcript isoforms and splicing events. Comparison of *cis-*spliced RNA fractions for each transcript (ΔPSI, per cent spliced in) between *mett-10^−/−^* and the wild-type animals identified, in total, 2456 differential *cis-*splicing events with a p-value < 0.05 or 1644 splicing events with an FDR < 0.05 (Figure 1C and E, Supplementary Table S2).

Out of 1644 *cis-*splicing events with an FDR < 0.05, the primary RNA splicing change in *mett-10^−/−^* animals was the alternative 5′SS usage (40.06%, Figure 1A and E), followed by alternative 3′SS usage (26.38%), exon skipping (13.31%), intron retention (12.34%) and alternative first exon usage (6.38%). In *A. thaliana*, the absence of FIO1 leads to alternative 5′SS usage and intron retention events at similar levels, followed by alternative 3′SS choice and exon skipping (33). Therefore, the absence of U6 snRNA m6A methyltransferase METT-10 in *C. elegans* leads to a stronger response in alternative 5′SS and 3′SS usage than other splicing events.

We next asked if trans-splicing was also affected in *mett-10^−/−^* animals. We quantified the trans-splicing defects using a new analysis pipeline using ONT-DRS reads. First, we annotated the SL splicing sites using Illumina and ONT-DRS data. Next, we counted reads containing the SL1 RNA or the outron sequence at the 5′-end of reads. Using this approach, we detected 563 significant outron retention events with a *P*-value <0.05 or 232 events with an FDR <0.05 (Figure 1D and F, Supplementary Tables S3 and S4). Of 563 outron retention events with a *P*-value <0.05, 58.44% were typical outron retained RNAs, and 25.75% were *cis-*spliced outron retained RNAs where the retained outron is *cis-*spliced, generating new exons. In addition, 15.81% of the outron retaining genes show alternative 3′ trans-splice site usage on the outron (Figure 1B and F).

In summary, using a combination of short and long-read sequencing, we show that loss of METT-10 function causes a wide range of *cis-* and trans-splicing defects in transcripts from more than 2000 genes.

### 5′ splice-sites with +4A are sensitive to loss of METT-10 function

**Figure 2.**
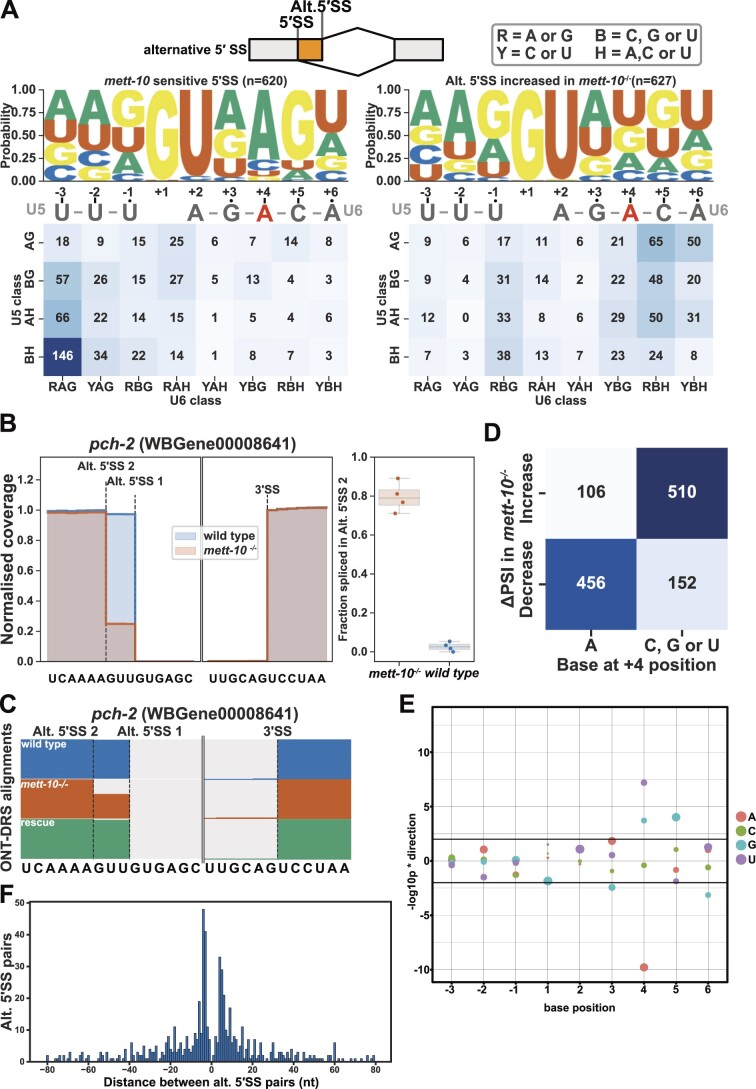
*mett-10* sensitive 5′SSs predominantly have +4A. (**A**) The sequence motif and frequency analysis of *mett-10* sensitive 5′SSs (−3 to + 6) and the alternative 5′SSs that are used more often in *mett-10^−/−^*. The sequence motif shows the probability of bases at each position around the 5′SS. U5 and U6 snRNA binding sequences are shown under the sequence motif logo. The frequency table is coloured based on the U5 snRNA interacting sequence frequency on the y-axis (−2 and −1) and U6 snRNA interacting sequence frequency on the x-axis (+3, +4 and + 5). (**B**) Normalised coverage of RNA-Seq reads for the *pch-2* intron 1 boundary. Alt. 5′SS 1 is used more often in wild-type animals and alt. 5′SS 2 is used more often in *mett-10^−/−^*. Barplot shows the fraction of reads supporting splicing at the Alt. 5′SS 2 over total reads in *mett-10^−/−^* and *wild-type* animals. (**C**) Normalised ONT-DRS alignments for the *pch-2* intron 1 boundary in wild-type, *mett-10^−/−^*, and *mett-10* germline rescued animals. (**D**) Heatmap showing the correlation between splice site usage in *mett-10^−/−^* (y-axis) and the specific base at position +4 of the 5′SS. (**E**) Effect size plot for the 5′SS positions −3 to + 6. Negative values indicate bases at the specific position are associated with significantly more alternative splicing, and positive values indicate bases at the specific position are associated with significantly less alternative splicing events. The circles' size corresponds to the base frequency at a given position across all 5′SSs in the genome (e.g. position 1 is predominantly G, and position 2 is predominantly U across all 5′SSs). (**F**) Histogram for the distance between alternative splice site pairs. The Y-axis shows the number of alternative splice site pairs, and the X-axis shows the distance between the pairs, with negative values indicating the alternative splice site moves upstream and positive values indicating the alternative splice site moves downstream of the original splice site.

To understand the basis of alternative 5′SS usage in *mett-10^−/−^* mutants, we compared the sequence features of sensitive 5′SSs for the positions −3 to + 6 (79). The majority of *mett-10^−/−^* sensitive 5′SSs have adenosine at the +4 position (+4A) within a //GURAG motif (Figure 2A, left panel). The alternative 5′SSs whose usage increased in *mett-10^−/−^* animals did not have +4A enrichment and were more likely to have an AG//GU consensus motif (Figure 2A, right panel, G-test *P*-value = 1.19e-168). Next, we compared the frequency of 5′SS base composition for the bases that interact either with the U5 snRNA (Figure 2A, U5 class −1 and −2) or the U6 snRNA (Figure 2A, U6 class +3, +4 and +5). The majority of the 5′SSs that were sensitive to the *mett-10* mutation had a non-AG consensus for the U5 snRNA interacting positions and an RAG motif for the U6 snRNA interacting sequence (Figure 2A, bottom left panel). In contrast, 5′SSs that were more often used in *mett-10^−/−^* animals were more likely to have an AG sequence for U5 snRNA interaction and a C, G or U at +4 position for U6 snRNA interaction (Figure 2A, bottom right panel). Therefore, we found a shift from 5′SSs with a //GURAG motif and a weak U5 snRNA interacting sequence selected in wild-type animals to 5′SSs that lack the //GURAG motif but have a stronger U5 interacting sequence selected in *mett-10^−/−^* animals.

The global switches in 5′SSs can be seen in individual genes. For instance, at *pch-2* (pachytene checkpoint 2), which encodes the *C. elegans* orthologue of human TRIP13 required for spindle checkpoint during mitosis and meiosis (80,81), exon 1 was exclusively spliced at the 5′SS UU//GUGAG in wild-type animals, whereas in *mett-10^−/−^* animals splicing occurred predominantly 3 nt upstream at the AA//GUUGU position (Figure 2B). As a result, PCH-2 amino acids Lys and Phe at positions 20 and 21 would be replaced with an Asn. We confirmed the alternative splicing of *pch-2* using orthogonal ONT-DRS data and found that the germline expression of *mett-10* in *mett-10^−/−^* background rescued *pch-2* 5′SS usage (Figure 2C). Similarly, at T12C9.7, which encodes the *C. elegans* orthologue of the human mitotic specific cyclin B2 (CCNB2), exon 8 was most frequently spliced at UU//GUGAG in wild-type and germline rescue animals. However, in *mett-10^−/−^* animals, the 5′SS choice moved to the nearby UG//GUUG (Supplementary Figure S2A; for additional examples, see Supplementary Figure S2A and S2B).

We found that 75% of all *mett-10* sensitive 5′SSs had +4A (Figure 2D). We calculated the effect size of each base on alternative splicing of 5′SSs between *mett-10^−/−^* and wild-type animals by comparing the distribution of ΔPSI values of SSs with or without a given base at each position (Figure 2E). 5′SSs with +4A had a significantly higher percentage of alternative splicing, and 5′SSs with +4G, +4U or + 5G were significantly less likely to be alternatively spliced (Figure 2E, *P*-value < 0.01). In addition, in *mett-10^−/−^* mutants, alternative 5′SSs were equally found upstream or downstream and primarily within ±5nt of the canonical splice site used in wild-type animals (Figure 2F).

In conclusion, 5′SS +4A is a key feature making 5′SSs sensitive to loss of *mett-10*, and the alternative 5′SSs used in the *mett-10^−/−^* mutants favour sequences without +4A and a stronger U5 interaction with the upstream exonic sequences.

### Loss of METT-10 function leads to intron retention and exon skipping

**Figure 3.**
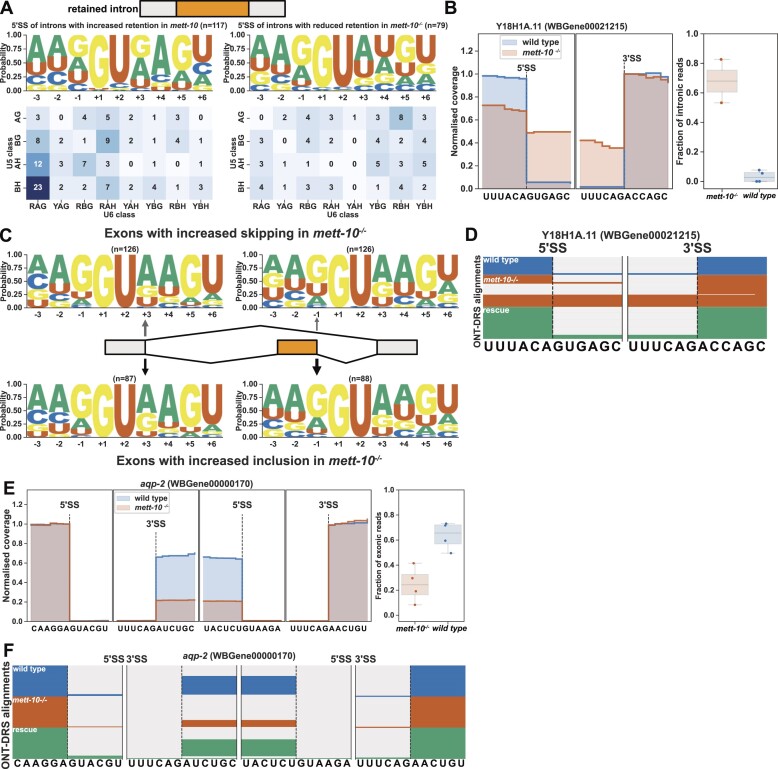
*mett-10^−/-^* animals have increased intron retention and exon-skipping. (**A**) 5′SS motif analysis of introns with increased (left) and decreased (right) retention in *mett-10^−/−^*. The frequency of sequences corresponding to U5 and U6 binding are shown in the heatmap. (**B**) Normalized RNA-Seq coverage of Y18H1A.11 intron 3 in *mett-10^−/−^* and *wild-type* animals. The bar plot shows the fraction of intronic reads over the retained intron. (**C**) 5′SS motif analysis of exons with increased skipping (upper panel) and increased retention (bottom panel) in *mett-10^−/−^* animals. Sequence motifs are shown for the upstream exon 5′SSs (grey) and the retained/skipped exon (orange). (**D**) Normalised ONT-DRS alignments for Y18H1A.11 intron 3 in wild-type, *mett-10^−/−^*, and *mett-10* germline rescued animals. (**E**) Normalized RNA-Seq coverage of *aqp-2* exon 5 in *mett-10^−/−^* and *wild-type* animals. Bar plots show the fraction of exonic reads over the skipped exon. (**F**) Normalized ONT-DRS alignments for *aqp-2* exon 5 in wild-type, *mett-10^−/−^*, and *mett-10* germline rescued animals.

Next, we investigated more closely the intron retention and exon skipping events observed in the absence of METT-10. We identified transcripts from 341 genes with *P*-value < 0.05 or 196 genes with an FDR < 0.05 that showed altered intron retention levels in *mett-10^−/−^* animals compared to wild-type animals (Supplementary Table S2). Out of 196 intron retention events with an FDR < 0.05, 117 showed increased intron retention, and 79 showed reduced intron retention (Figure 3A). The 5′SSs of introns with increased retention in *mett-10* mutants had the //GURAG motif and weak U5 recognition sequence (Figure 3A, left panel). In contrast, the 5′SSs of introns with reduced retention had the AG//GU motif and lacked +4A (Figure 3A, right panel, G-test *P*-value = 2.9e-11). For example, at *C. elegans* gene Y18H1A.11, which encodes a choline-phosphate cytidylyltransferase, the *C. elegans* orthologue of human PCYT1, the 5′SS has CA//GUGAG sequence, which conforms to the //GURAG motif and a weak U5 interacting CA dinucleotide (Figure 3B). Y18H1A.11 showed significant intron retention in *mett-10* mutants (Figure 3B and D, *P*-value < 0.05), and intron retention was rescued with germline *mett-10* expression (Figure 3D).

Next, we compared the 5′SS sequences of introns adjacent to the exons that showed increased skipping or inclusion in *mett-10*^−/−^ animals compared to the wild-type (Figure 3C). Exons that showed increased skipping had a 5′SS //GURAG motif with a higher probability of +4A compared to the exons that showed reduced skipping, which had a higher probability of AG//GU (Figure 3C). However, the overall consensus motif between −3 and + 6 positions was not significantly different (G-test *P*-value = 0.47). This could be due to multiple splice sites influencing the exon-skipping events. The exon 5 of *aqp-2*, the orthologue of the human Aquaporin3, was frequently skipped in *mett-10^−/−^* animals compared to wild-type and *germline rescue* animals (Figure 3E and F). The 5′SS adjacent to the *aqp-2* exon 5 has the CU//GUAAG sequence that fits the //GURAG motif and a weak U5 interacting CU dinucleotide. Therefore, intron retention and exon skipping events observed without METT-10 function predominantly involve 5′SSs with a +4A.

### Loss of METT-10 function affects 3′SS usage

**Figure 4.**
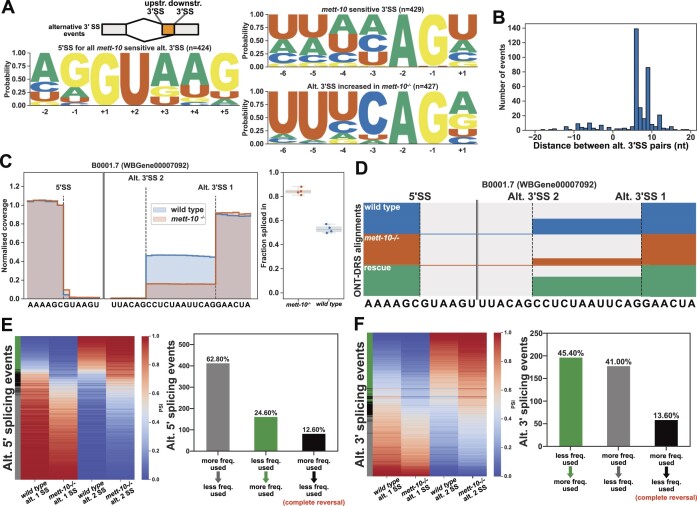
The absence of *mett-10* affects 3′SS usage. (**A**) 5′ and 3′SS motif analysis of transcripts that are *mett-10* sensitive (upper panel) and the corresponding alternative 3′SSs whose usage increases in *mett-10^−/−^* (bottom panel). (**B**) Histogram for the distance between alternative splice site pairs. The Y-axis shows the number of alternative splice site pairs, and the X-axis shows the distance between the pairs. Negative values indicate the alternative splice site moving upstream, and positive values indicate the alternative splice site moving downstream of the original splice site. (**C**) Normalized RNA-Seq coverage of B0001.7 intron 6. The canonical splice position is Alt. 3′SS 1. In wild-type animals, weak upstream splice site Alt. 3′SS 2 is also utilized. The bar plot shows the fraction of reads covering the exon sequence to the right of the canonical splice site. (**D**) Normalized ONT-DRS alignments for B0001.7 intron 6 in wild-type, *mett-10^−/−^* and *mett-10* germline rescued animals. (**E**) Heat map (left) and bar plots (right) showing the frequency of 5′SS usage at specific splice sites in wild-type and *mett-10^−/−^* animals. (**F**) Heat map (left) and bar plots (right) showing the frequency of 3′SS usage at specific splice sites in wild-type and *mett-10^−/−^* animals.

The second most abundant class of *cis-*splicing changes observed in the absence of the METT-10 function is alternative 3′SS usage (Figure 1E). In *mett-10^−/−^* animals, we observed 656 alternative 3′SS usage events with a *P*-value <0.05, of which 434 had an FDR < 0.05 (Supplementary Table S2). *mett-10* sensitive 3′SSs do not have the conserved *C. elegans* 3′SS motif of UUUCAG//R (which is required for efficient U2AF65-U2AF35 binding). In contrast, the 3′SSs with increased usage in *mett-10^−/−^* have a strong UUUCAG/R motif (Figure 4A, G-test −6 to +1 positions *P*-value = 2.7e-149).

Most of the alternative 3′SS usage was observed at introns where the selected 5′SSs had a +4A (263) (Supplementary Figure S3A), suggesting that 3′SS usage was linked to 5′SS choice. Alternative 3′SSs used in *mett-10^−/−^* animals were predominantly found downstream of the canonical 3′SS (Figure 4B). We found that most *mett-10^−/−^* sensitive 3′SSs (365) involved a switch in usage from a weak upstream 3′SS sequence to a strong downstream 3′SS (Supplementary Figure S3B, bottom panels). The remaining alternative 3′SS events (64) reflected shifts in usage from a stronger downstream 3′SS to a moderate upstream 3′SS, where the + 1R and -3C frequencies were lower than the 3′SSs shifting downstream (Supplementary Figure S3B, top panels). For example, in wild-type animals, intron 6 of the B0001.7 gene was frequently spliced at the downstream 3′SS AUUCAG//G and, to a lesser extent, at the upstream 3′SS UUACAG//C (Figure 4C and D). In *mett-10* mutants, most of the splicing events occurred at the downstream 3′SS and the frequency of splicing at the upstream 3′SS was significantly reduced (Figure 4C and D). Similarly, in wild-type animals, intron 4 of *pdk-1*, which encodes a conserved protein kinase with a role in organismal sterility, was frequently spliced at the downstream 3′SS UUUCAG//A and the upstream 3′SS AGAAAG//U (Supplementary Figure S2C). In *mett-10* mutants, the frequency of splicing events at the upstream 3′SS was significantly reduced, and most splicing events happened at the downstream 3′SS (Supplementary Figure S2C). The germline expression of *mett-10* rescued the upstream 3′SS usage in intron 6 of B0001.7 and intron 4 of *pdk-1*, similar to wild-type levels (Figure 4D and Supplementary Figure S2C).

In contrast to shifts in alternative 5′SS usage in *mett-10* mutants from a site used more frequently in wild-type animals to a splice site used less frequently, alternative 3′SS usage shifted from a splice site used less frequently in wild-type animals to a splice site used more frequently in wild-type animals (Figure 4E and F). For the alternative 5′ and 3′SS usage observed in *mett-10* mutants, 12.6% of the 5′SS events and 13.6% of the 3′SS events showed a complete reversal of the splice site usage from a more frequently used splice site in wild-type animals to a less frequently used splice site in wild-type animals (Figure 4E and F, black bars vs grey bars).

We observed similar intron retention and alternative 3′SS usage in *sams-3 and -4* except for *sams-5*, but in all cases, the amplitude of the intron retention and alternative 3′SS usage in wild-type animals were smaller than previously reported (35,41) (Supplementary Figure S4A and S4B). Of all the alternative 3′SS usage events that shift from an upstream position to the downstream canonical position (365), only 3.8% (14) have the methylation motif UACAG//A at the canonical splice site.

ONT-DRS data can predict RNA modifications (45,82–85). We used a signal-level analysis approach, *Yanocomp* (45), and a differential error rate approach, *differ* (50) to predict the modified bases in our ONT-DRS data. Our analysis of the ONT-DRS reads comparing the *mett-10* mutant and wild-type animals and using two different approaches did not reveal any METT-10-dependent m6A modification at the *sams-3, -4* and *-5* intron sequences (Supplementary Tables S5 and S6).

### 3′ *trans*-Splice site sequence features determine SL trans-splicing sensitivity to loss of *mett-10*

**Figure 5.**
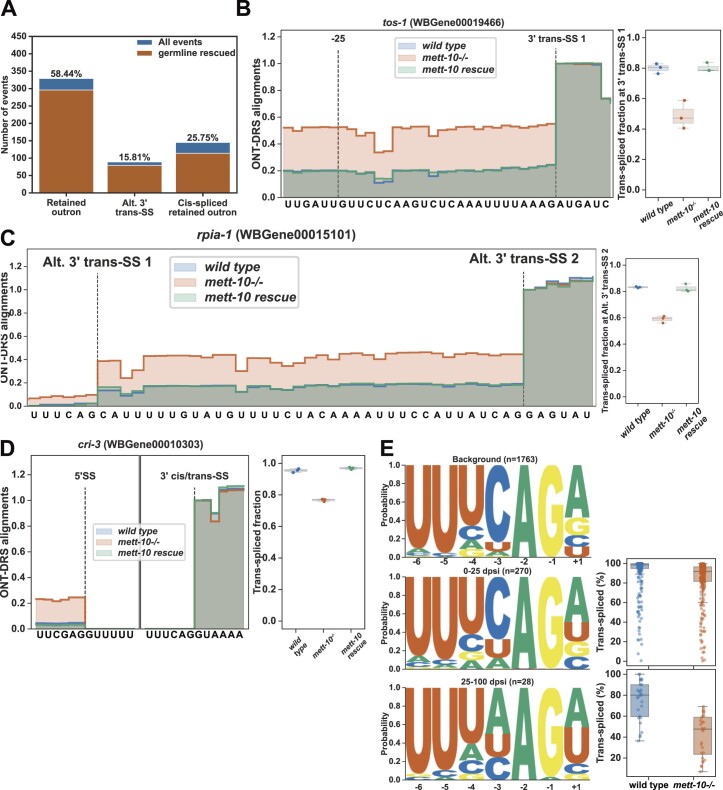
*mett-10* is required for efficient trans-splicing. (**A**) The trans-splicing defects rescued by the germline expression of *mett-10* are shown in orange over the total SL trans-splicing defects observed in *mett-10^−/−^* animals as in Figure 1F (blue). (B–D) Examples for the (**B**) outron retention, (**C**) alternative 3′ trans-splice site usage, (**D**) *cis-*spliced outron retention events showing the normalised coverage of ONT-DRS alignments (left) and the fraction of reads covering the outron sequence (right) in wild-type, *mett-10^−/−^* and *mett-10* germline rescued animals. (**E**) 3′ trans-splice site motif of background transcripts that do not show the trans-splicing defect (top panel), transcripts that show the weak trans-splicing defect (middle panel) and transcripts that show the strong trans-splicing defect (bottom panel).

In addition to *cis-*splicing defects, we observed significant SL trans-splicing defects in *mett-10^−/−^* animals using ONT-DRS (Figure 1F). We found that the germline expression of *mett-10* can rescue most of the trans-splicing defects found in *mett-10^−/−^* (Figure 5A–D). Normalized coverage of ONT-DRS alignments show that all trans-splicing classes; outron retention (Figure 5B), alternative 3′ trans-splice site usage (Figure 5C) and *cis-*spliced retained outron (Figure 5D) are effectively rescued.

In *mett-10^−/−^* animals, although hundreds of genes have trans-splicing defects, most of the RNAs are still effectively trans-spliced. Therefore, we hypothesised that there could be sequence determinants of *mett-10^−/−^* trans-splicing sensitivity at the 3′ trans-splice site sequences rather than the invariable 5′ trans-splice sites. We grouped the transcripts according to the extent of the trans-splicing difference (ΔPSI) and analysed the 3′ trans-splice site consensus motifs separately (Figure 5E and Supplementary Figure S5A). Compared to the 3′ trans-splice site motif of a background set of effectively trans-spliced genes, genes that show trans-splicing defects in *mett-10^−/−^* had weaker 3′ *trans*-splice site sequences (Figure 5E and Supplementary Figure S5A). Genes with lower ΔPSI had 3′ *trans*-splice site motifs with minor deviations from the background motif, particularly affecting the −4U, −3C and +1R (Figure 5E, middle panel and Supplementary Figure S5A). Genes with higher ΔPSI showed the most significant variation from the conserved UUUCAG/R motif, mainly at the −4U, −3C and +1R (Figure 5E, bottom panel and Supplementary Figure S5A). We analysed the frequency of U2AF65 and U2AF35 binding sequences at the 3′ trans-splice sites of the control sequences and the sequences that showed significant ΔPSI (Supplementary Figure S5B). We found that both U2AF65 and U2AF35 recognition motifs deviated from the consensus UUUCAG/R motif, with the U2AF35 recognition sequence showing the largest deviation (G-test *P*-value 2.78 × 10^−6^, Supplementary Figure S5B).

In summary, without *mett-10*, *C. elegans* pre-mRNAs with weak 3′ trans-splice sites fail to trans-splice efficiently and accurately.

### Consequences of *cis-* and *trans*-splicing defects in coding potential of transcripts

To investigate the impact of alternative splicing in *mett-10^−/−^* on protein translation, we analysed the change in open reading frames (ORFs) of alternatively spliced transcripts. We analysed all *cis-*splicing events for their 3 nt periodicity and categorized the events in-frame if the change is a multiple of 3 nt and out-of-frame if the change is not a multiple of 3 nt. Most intron retention events created out-of-frame changes (61.1%), and most exon-skipping events created in-frame changes (65.8%, Supplementary Figure S5C). Most alternative 5′SS events are out of frame (55.4%), whereas most 3′SS events are in-frame (74.9%, Supplementary Figure S5C). For instance, the alternative 5′SS in the neuronal transcript Y41C4A.12 created an early termination codon truncating the open reading frame by 23 amino acids (Supplementary Figure S5F). *C. elegans* C55A6.10 encodes a protein homologous to the human C12orf4, linked to autosomal recessive intellectual disability (86). The alternative 5′SS in transcripts of C55A6.10 created an early termination codon in the middle of the gene (Supplementary Figure S5G).

For trans-splicing events, we specifically looked for potential upstream open reading frames that can arise due to outron retention or the *cis-*splicing of retained outrons. For outron retained and *cis-*spliced retained outron events, we used the most extended ONT-DRS reads as representatives of the 5′ end of transcripts. In most cases, these reads will likely be shorter than the actual transcription start sites (50). Next, we searched for the ATG start codon within outron regions or the *cis-*spliced retained outron regions. We found that 61.3% of all retained outron regions and 91.8% of all *cis-*spliced retained outrons contain at least one start codon sequence (Supplementary Figure S5D). In both outron retention classes, most potential upstream start codons are in-frame with the downstream canonical start codon (60.4% of retained outrons and 82.9% of *cis-*spliced retained outrons, Supplementary Figure S5D). The percentages of in-frame start codons within outron regions of outron-retaining genes are higher than in-frame start codons observed in all SL spliced non-outron retaining genes when considering a median outron size of 120nt (Supplementary Figure S5E).

In addition, to start codons, eukaryotic translation initiation generally requires a Kozak sequence (87). We analysed the sequence composition of all *C. elegans* annotated start codons from positions −4 to +4 and confirmed that the ideal Kozak sequence of AAA (88,89) is present in only 16% of *C. elegans* start codons (Supplementary Figure S5H and S5I). We then analysed the presence of an ideal *C. elegans* Kozak sequence and its derivatives in outron-retained genes and *cis-*spliced outron-retained genes and compared them to events that we previously identified as being in-frame. Our analysis showed little difference between any subset of start codons, but the in-frame *cis-*spliced retained outrons had the most frequent AAA Kozak sequence (Supplementary Figure S5H and S5I).

We conclude that *cis-* and trans-splicing defects in *mett-10^−/−^* animals cause large-scale in- and out-of-frame changes to coding transcripts. In addition, upstream start codons which emerge due to trans-splicing defects are indistinguishable from the canonical start codons and could function in protein translation.

### 5′SSs with +4A are sensitive to mutations in METT-10 and the spliceosomal protein SNRNP27K

**Figure 6.**
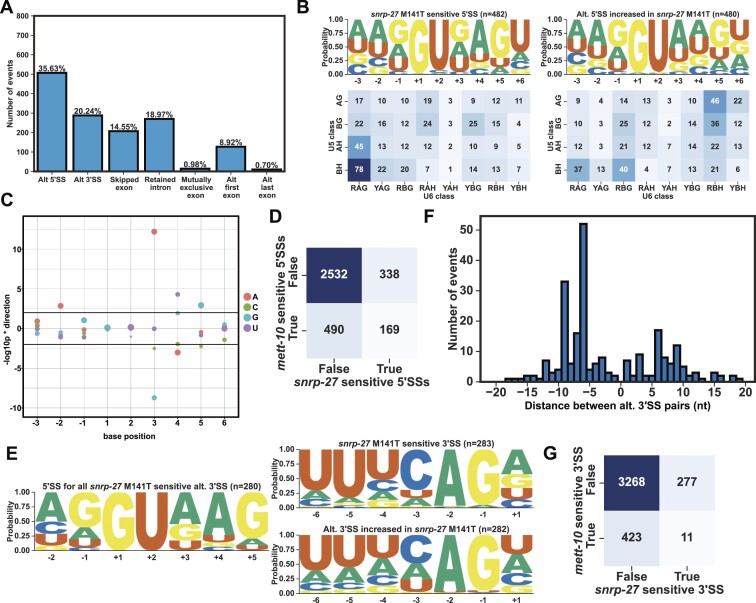
*snrp-27* is required for the efficient splicing of 5′SSs with +4A. (**A**) Classification of all significant (FDR < 0.05) *cis-*splicing defects in *snrp-27(az26)* M141T animals compared to CB936 (*unc-73*(e936)). (**B**) Sequence motif (upper panels) and the frequency of U5 and U6 interacting sequences (bottom panels) of the 5′SSs that are either sensitive to *snrp-27(az26)* (left panels) or used more often in *snrp-27(az26)* (right panels). (**C**) Effect size plot for the 5′SS positions −3 to + 6. Negative values indicate bases at the specific position are associated with significantly more alternative splicing, and positive values indicate bases at the specific position are associated with significantly less alternative splicing events. The circles' size corresponds to the base frequency at a given position across all 5′SSs in the genome. (**D**) Heat map showing the overlap of *mett-10* and *snrp-27* sensitive 5′SSs. Row and column names ‘true’ and ‘false’ refer to whether these rows and columns fulfil the condition in the axis names. The expected overlap between *mett-10* and *snrp-27* is 95, and the observed overlap is 169, *P*-value = 5.3e-20. (**E**) Sequence motif analysis of 3′SSs that are either sensitive to *snrp-27(az26)* (upper panel) or used more often in *snrp-27(az26)* (bottom panel). 5′SS motif of sensitive 3′SSs is shown on the left. (**F**) Histogram showing the distance between alternative 3′SS pairs (x-axis) and the number of 3′SS events (y-axis) (**G**) Heat-map showing the overlap of *mett-10* and *snrp-27* sensitive 3′SSs. Row and column names ‘true’ and ‘false’ refer to whether these rows and columns fulfil the condition in the axis names. The expected overlap between *mett-10* and *snrp-27* is 32, and the observed overlap is 11, *P*-value = 7.14 × 10^–5^.

Structural studies of spliceosomal complexes have not fully resolved how U6 snRNA m6A interacts with the 5′SS because the methyl group is not yet resolved in available cryo-EM models of the spliceosome complexes. A recent phylogenetic analysis identified a strong association between METTL16, the spliceosomal protein SNRNP27K (*snrp-27* in *C. elegans*) and the 5′SS +4A (90). Consistent with this, M141T mutation in SNRP-27 leads to alternative splicing of 5′SSs with +4A (72). To understand if METT-10 and SNRP-27 influence the splicing of the same 5′SSs in *C. elegans*, we re-analysed the previously published RNA-Seq data from *snrp-27(az26)* animals that carry the M141T mutation and the corresponding parental line (CB936 *unc-73*(e936)) (72) using our RNA splicing analysis pipeline. We identified more alternative splicing events (2159) than previously reported. The most abundant event class was alternative 5′SS usage (Figure 6A). Like *mett-10^−/−^*, *snrp-27(az26)* mutation led to reduced splicing at 5′SSs with +4A and a //GURAG motif, and splice site usage switched more frequently to alternative 5′SSs with an AG//GU motif (G-test *P*-value 5.07 × 10^−39^, Figure 6B). 60.7% of all 5′SSs with a reduced usage in *snrp-27(az26)* had +4A (Supplementary Figure S6A). The effect size comparison of each base, from −3 to + 6 position, between *snrp-27(az26)* and wild-type animals showed that 5′SSs with + 3G and +4A have a significantly higher percentage of alternative splicing in *snrp-27(az26)* and 5′SSs with −2A, +3A, +4U or + 5G were significantly less likely to be alternatively spliced in *snrp-27(az26)* (Figure 6C). Similar to *mett-10* mutants, in *snrp-27(az26)*, alternative 5′SSs could be found upstream and downstream of the canonical wild-type splice site in *snrp-27(az26)* (Supplementary Figure S6B).

There was a significant overlap between the *mett-10* and *snrp-27* sensitive 5′SSs (Figure 6D, the expected overlap is 95, and the observed overlap is 169, *P*-value = 5.3e-20). Although 5′SSs sensitive to either one or both proteins shared a //GURAG motif, 5′SSs sensitive to both *mett-10* and *snrp-27* had a more dominant //GUGAG motif (Supplementary Figure S6C). In contrast, 5′SSs that were only sensitive to *mett-10* had a //GUAAG motif (Supplementary Figure S6D), and the 5′SSs that were only sensitive to *snrp-27* had a //GUDAG motif (D = A, G or U) (Supplementary Figure S6E). Therefore, in addition to the +4 position, the +3 position of 5′SSs is important for effective and accurate splicing mediated by SNRP-27.

Unlike *mett-10* sensitive 3′SSs that were defined by a weak 3′SS motif, *snrp-27* sensitive 3′SSs have a strong UUUCAG/R motif, and the alternative 3′SSs are weaker, despite having a similar //GURAG motif at their 5′SSs (G-test *P*-value 9.93 × 10^−17^, Figure 6E). In contrast to the alternative 3′SS usage in *mett-10^−/−^*, most of the alternative 3′SS events in *snrp-27(az26)* were found upstream of the canonical wild-type site (Figure 6F). Such changes in 3′SS usage involved a shift from a strong UUUCAG/R 3′SS to a weak 3′SS motif (Supplementary Figure S6F). These differences between *mett-10* and *snrp-27* sensitive 3′SSs were reflected in the lack of overlap in 3′SSs sensitive to the absence of either gene function (Figure 6G, the expected overlap is 32, and the observed overlap is 11, *P*-value = 7.14 × 10^–5^).

In summary, METT-10 and SNRP-27 are required for accurate and efficient splicing of many 5′SSs with +4A. METT-10 and SNRP-27 affect an overlapping subset of genes, but differences in the +3 position distinguish the sensitivity of some 5′SSs. In contrast, METT-10 and SNRP-27 affect distinct 3′SSs.

### METTL16 and THUMPD2 influence the selection of distinct 5′SSs in human cells

**Figure 7.**
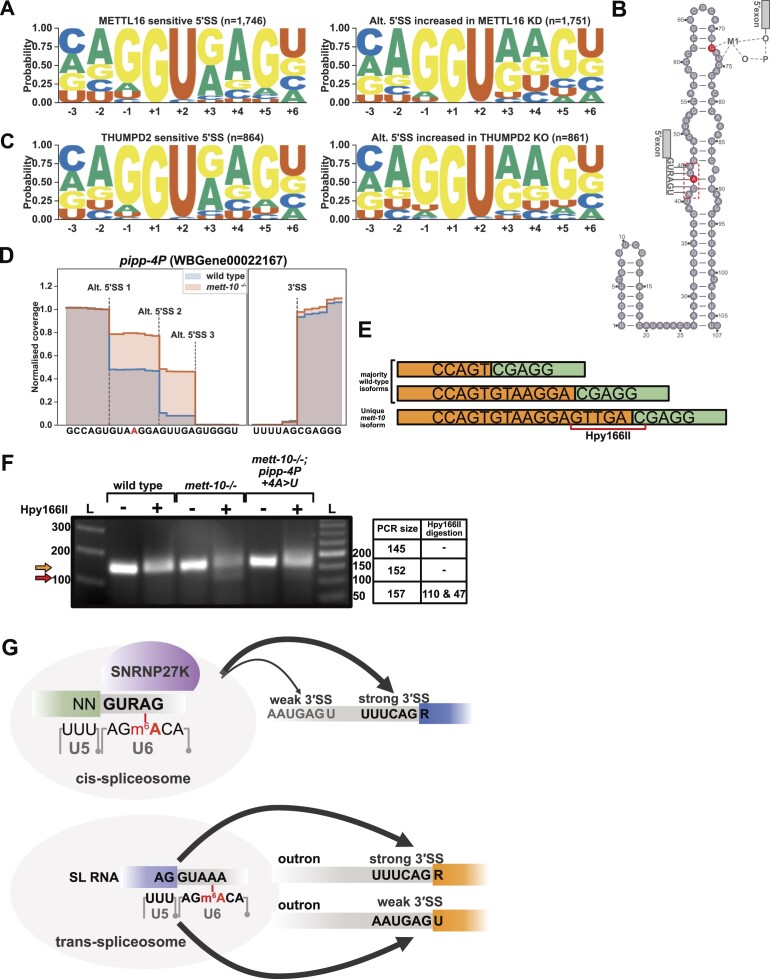
Human METTL16 is required for efficient splicing of 5′SS with +4A, and *in vivo*, editing of 5′SS +4A to +4U can restore splicing in *mett-10^−/−^* mutants. (**A**) Sequence motif analysis of METTL16 sensitive 5′SSs (left) and 5′SSs whose usage increases in METTL16 knock-down cells (right). The p-value for the comparison of motifs is 9.35 × 10^−156^. (**B**) Human U6 snRNA showing the positions of m6A and m2G methylations in red. The UACAGA box is highlighted with a red dashed line. Grey boxes indicate 5′ exons and depict interactions between m6A43 and m2G72 with the 5′SSs. M1 depicts the catalytic metal ion. (**C**) Sequence motif analysis of THUMPD2 sensitive 5′SSs (left) and 5′SSs whose usage increases in THUMPD2 knock-out cells (right). The p-value for the comparison of motifs is 0.98. (**D**) Normalized read coverage of *pipp-4P* intron 4 5′SSs. In wild-type animals, splicing was observed most frequently at Alt. 5′SS 1 position followed by Alt. 5′SS 2 position. In *mett-10* mutants, splicing was observed most frequently at Alt. 5′SS 3 position followed by Alt. 5′SS 2 position. Adenosine position edited to uracil is shown in red. (**E**) cDNA visualisation of three splice isoforms for *pipp-4P* exon 4 and 5. Orange depicts the upstream exon, and green depicts the downstream exon. The hpy166I restriction enzyme recognition sequence is shown by the red bar. (**F**) Gel electrophoresis analysis of *pipp-4P* RT-PCR products followed by Hpy166I digestion. The orange arrow shows the undigested product size, and the red arrow shows the digested product size. The table shows the expected product sizes for RT-PCR and digestion. (**G**) Model for the function of U6 snRNA m6A modification in 5′SS recognition, alternative splicing of 3′SS and trans-splicing. m6A methylated U6 snRNA facilitates accurate recognition of 5′SSs with a //GURAG motif through non-canonical base pairing with +4A (Top panel). SNRNP27K likely functions together with m6A methylated U6 snRNA to recognize 5′SSs with +4A. Efficient recognition of 5′SSs can facilitate alternative splicing at weak 3′SSs. Similarly, efficient recognition of SL RNA 5′SSs by m6A methylated U6 snRNA can facilitate trans-splicing at weak 3′ *trans*-splice sites.

Since the impact of the loss of *C. elegans* METT-10 function on splicing resembled that of the corresponding mutants in *S. pombe* and *A. thaliana*, we next asked if a similar impact occurred in human cells with disrupted METTL16 function. We re-analysed the previously published RNA sequencing data from human control 293A-TOA cells (HEK293 derivative Tet-off advanced cells) and cells subjected to METTL16 knock-down (31). We found that METTL16 knock-down leads to the mis-splicing of thousands of pre-mRNAs, with exon skipping and intron retention being the most prominent alternative splicing events (Supplementary Figure S7A). Sequence motif analysis of 1746 METTL16 sensitive 5′SSs showed a strong enrichment for +4A (G-test *P*-value 9.35 × 10^−156^, Figure 7A and Supplementary Figure S7B, left panels) compared to alternative 5′SSs whose usage increased upon METTL16 knock-down, where the +4 position was more likely to be non-A bases (Figure 7A and Supplementary Figure S7B, right panels). This shift in +4A preference could also be seen in the sequence frequency of the U6 interacting bases, along with a shift towards stronger U5 interacting bases (Supplementary Figure S7B). However, human 5′SSs generally appeared to have stronger U5 interacting sequences (Supplementary Figure S7B).

As a control, we next compared the impact of a different U6 modification to that made by METTL16. U6 snRNA m2G72 modification by the methyltransferase THUMPD2 is important for pre-mRNA splicing (29,30). While U6 snRNA m6A43 resides within the UACAGA box that base pairs with the 5′SS, m2G72 interacts with the catalytic metal ion M1 within the catalytic centre of the spliceosome (91–93) (Figure 7B). We re-analysed the RNA sequencing data that was previously generated from wild-type HCT116 cells and THUMPD2 knock-out cells (29). Our analysis confirmed the large-scale disruption of pre-mRNA alternative splicing upon loss of THUMPD2 function (Supplementary Figure S7C). However, the sequence motif analysis of THUMPD2 sensitive 5′SSs did not show +4A preference, and there was no clear difference between the 5′SS motif of sensitive sites and the sites whose usage increased in THUMPD2 knock-out (G-test *P*-value 0.98, Figure 7C and Supplementary Figure S7D).

We conclude that METTL16’s function in pre-mRNA splicing is conserved between *S. pombe, A. thaliana, C. elegans* and humans. The sensitivity of 5′SSs with +4A is specific to the absence of the m6A methyltransferase METTL16 and not seen in the absence of another U6 snRNA methyltransferase important in pre-mRNA splicing, revealing that these modifications have distinct functions within the spliceosome.

### Editing endogenous 5′SS +4A to +4U can restore wild-type splicing in *mett-10^−/−^* animals

Our analysis showed that METT-10 and METLL16 sensitive 5′SSs predominantly had a +4A, and the alternative 5′SS positions were relatively enriched for non-A bases, especially +4U (Figures 2 and 7). To get a mechanistic understanding of how U6 snRNA m6A interacts with the 5′SS +4A, we tested if replacing +4A sites with +4U could rescue splicing in *mett-10^−/−^* mutants. We used targeted CRISPR/Cas9 mutagenesis in *mett-10^−/−^* animals to edit +4A positions in 5′SSs sensitive to the absence of METT-10. We successfully edited the 5′SS of *pipp-4P* intron 4 (Y71H2AM.2, WBGene00022167) from a +4A position to a +4U (Supplementary Figure S7E). *pipp-4P* encodes the *C. elegans* orthologue of human phosphatidylinositol (4,5) bisphosphate phosphatase, and in wild-type animals, *pipp-4P* intron 4 was predominantly spliced at two alternative 5′SS positions with most frequent splicing at a GU//GUAAG position (Figure 7D). In *mett-10* mutants, *pipp-4P* was predominantly spliced at a third, distal, 5′SS position (Figure 7D and E). The distal splice site used most frequently in *mett-10^−/−^* contains a Hpy166I restriction site that can detect the mutant isoform following RT-PCR and restriction digestion (Figure 7E, F, Supplementary Figure S7F, S7G and S7I). We observed that in *mett-10^−/−^* animals where we replaced the +4A to +4U within the *pipp-4P* intron 4 alternative 5′SS 1 position, RT-PCR products with the Hpy166I restriction site were strongly reduced or absent without changing the overall PCR size (three biological replicates, Figure 7F, Supplementary Figure S7F, S7G, S7H and S7I).

Our results show that converting +4A to +4U in *mett-10* sensitive 5′SSs can restore wild-type splicing in *mett-10* mutants that lack U6 snRNA m6A modification.

## Discussion

### The absence of METT-10 in *C. elegans* and METTL16 in humans leads to a wide range of splicing defects at 5′SSs with +4A

We identified that, in *C. elegans*, the absence of *mett-10* causes large-scale *cis-* and *trans*-splicing defects (Figure 1C–F) consistent with the function of METT-10 in m6A methylation of U6 snRNA. Previously, it was suggested that *mett-10* mutants do not show global splicing defects (35). We were able to capture the *cis-* and *trans*-splicing changes by deep sequencing of Illumina libraries in quadruplicate, using ONT-DRS, and building condition-specific reference transcriptomes. Our results show that the pre-mRNAs of thousands of genes are mis-spliced in *mett-10* mutant animals, and many of these events are likely to contribute to the developmental and germline phenotypes of *mett-10* mutants.

We further show that, in *C. elegans* and human cells, the absence of METT-10/METTL16 specifically affects 5′SSs with a //GURAG motif, and the 5′SS +4A is a major determinant of splice site usage (Figures 2A and 7A). In the absence of METT-10/METTL16, splice site usage shifts from a //GURAG motif to an AG//GU motif, usually favouring +4U. These features of alternative splice site usage are also conserved in *A. thaliana* FIO1 mutants for 5′SSs and *S. pombe* MTL16 mutants for intron retention events (33,36). Our data and the data from previous studies on yeast and plants align with the function of METT-10/METTL16 as a major regulator of pre-mRNA splicing through U6 snRNA m6A modification. U6 snRNAs with m6A43 (m6A38 in *C. elegans*) potentially interact more strongly with the 5′SSs with +4A through a *trans*-Hoogsteen sugar edge interaction between m6A43:+4A (Supplementary Figure S8C and S8D) (5,33,36,94–96). The increased thermostability of m6A:A over A:A was shown *in vitro* between the ACAGA box of U6 and an intron sequence (36). Without m6A43, 5′SS usage moves to sequences that support more robust U5 snRNA recognition, such as AG//GU, a stronger unmethylated U6 snRNA recognition, such as with +4U, or both (further discussed in (33,36,90)). Consequently, spliceosomes abort splicing altogether where a suitable 5′SS is not found, as in intron retention and exon-skipping events. Indeed, the absence of METT-10 and likely U6 snRNA m6A modification leads to intron retention and exon-skipping where 5′SS //GURAG motif and +4A are essential (Figure 3A–C). Importantly, our analysis shows that the presence of +4A can distinguish 75% of all alternative 5′SS events, suggesting a direct role for *mett-10* and U6 snRNA m6A modification in splicing (Figure 2D). In addition, we did not observe any correlation between splicing changes and steady-state gene expression in *mett-10^−/−^* animals (Supplementary Figure S8A and S8B). We also showed that the 5′SS +4A preference is specific to U6 snRNA m6A modification as the mutation of the U6 snRNA m2G methyltransferase THUMPD2 does not affect 5′SS usage in a sequence-dependent manner (Figure 7C).

In further support of our model where U6 snRNA m6A modification is required for efficient recognition of 5′SS +4A positions, we edited an endogenous 5′SS +4A to a +4U in *mett-10^−/−^* mutant animals and restored splicing to wild-type splice site positions (Figure 7F). For base-pairing interactions, A:U would be more favourable to m6A:A. Therefore, by converting a 5′SS sequence that was a better base pairing substrate for the m6A-modified U6 snRNA ACAGA to a 5′SS sequence that is a better base pairing substrate for the unmodified U6 snRNA ACAGA, we were able to change the accuracy and efficiency of splicing.

Our analysis suggests that in *C. elegans* and humans, U5 and U6 snRNA base pairing is critical for 5′SS selection, but most alternative 5′SSs remain in close proximity to the canonical 5′SSs. The alternative 5′SSs used in *A. thaliana* FIO1 mutants show a similar distribution (Parker et al. 2022), suggesting that U1 snRNA binding could limit the final position of the transesterification reaction decided by U6 snRNA. Alternatively, U1 snRNA could bind to multiple positions, and the strength of U6 snRNA binding could favour one 5′SS over another (97). Several studies support a model whereby U1 base pairing at one position can lead to the use of multiple 5′SSs through U6 base pairing (98,99), whereas other studies support a requirement for the base pairing of U1 and U6 at the same 5′SS (100,101). Our results show that for 5′SSs with a //GURAG motif, U6 snRNA m6A modification status is a decisive factor for the final 5′SS position.

We observed a compensatory pattern between the base composition of U5 and U6 snRNA interacting sequences. A similar compensatory ‘seesaw linkage’ pattern was observed for the base composition of human 5′SSs where −1G allows any nucleotide at +5 position, and +5G allows any nucleotide at −1 position (102–105). Later, it was shown that having a G at both −1 and + 5 positions allowed more efficient splicing (106). The two classes of 5′SSs, AG//GU and //GURAG, identified by U5 and U6 snRNA binding, could account for such compensatory base compositions at 5′SSs (33).

### METT-10 and SNRNP27K functionally interact for accurate splicing of 5′SSs

Available structures of the spliceosome suggest that the spliceosomal protein SNRNP27K is positioned close to the U6 snRNA and could function in 5′SS recognition (Supplementary Figure S8C) (5). Recent re-analysis of the cryo-EM structures of the human spliceosome suggests that M141 of SNRNP27K is positioned close to the space that becomes occupied by the m6A43 of U6 snRNA (90). Our analysis of the available RNA-Seq data from SNRP-27 M141T mutants strongly supports a functional interaction between METT-10 and SNRP27K in splicing 5′SSs with +4A (Figure 7G). This could happen through SNRNP27K functioning to position U6 snRNA correctly during B-complex formation.

### METT-10 and SNRNP27K regulate distinct 3′SSs

The effect of METT-10 and SNRP-27 on alternative usage of 3′SSs is distinct. In the absence of METT-10, 3′SS usage shifts from weak upstream 3′SSs to downstream strong canonical 3′SSs, suggesting that METT-10 and likely U6 snRNA m6A modification are important for the stabilization of 5′ and 3′SS interactions (Figure 7G). Our data indicates that, in *C. elegans*, this interaction could depend on the strength of 3′SS sequences primarily based on U2AF binding motifs (Figure 4A). We identified that, in the absence of *mett-10*, SL trans-splicing also fails at weak 3′ trans-splice sites based on U2AF consensus motifs (Figure 5E).

In the absence of SNRP-27, usage of strong downstream 3′SSs shifts to weaker upstream positions, suggesting that in the absence of SNRP-27, distance of the 3′SS to the 5′SS could be a critical factor and SNRP-27 could function to facilitate distal 3′SS selection. U6 snRNA interacts with both the 5′SS and 3′SS during the C to C* transition of the spliceosome (107) (discussed for the *A. thaliana* alternative 3′SS usage (33)), and this interaction can explain why U6 snRNA m6A is important for 3′SS selection. Indeed, many spliceosomal proteins associated with the C* complex affect 3′SS usage (108). However, SNRNP27K is no longer present in human C and C* complexes. The impact of SNRP-27 on orienting the 5′SSs for U6 snRNA binding in *C. elegans* may extend beyond the time it is present in the spliceosome.

In *C. elegans*, similar shifts in alternative 3′SS usage events have been observed in tissue-specific gene expression (109) and during ageing (110). Therefore, U6 snRNA m6A modification and SNRP-27 could have regulatory roles during animal development.

### The diversity of 5′SS sequences suggests U6 snRNA m6A methylation could be a regulated process

Overall, our analysis of splicing events observed in *mett-10^−/−^* and wild-type animals suggests there are two classes of 5′SSs in *C. elegans*: 5′SSs with the //GURAG motif and +4A, which prefer U6 snRNAs that are m6A methylated and the 5′SSs with AG//GU or +4U that would prefer unmethylated U6 snRNAs. Our experimental data is consistent with the previous *in silico* analysis of annotated *C. elegans* 5′SSs (33). Indeed, within all *C. elegans* 5′SS sequences (Supplementary Figure S8E), 44% of the 5′SSs have a //GURAG motif (Supplementary Figure S8F), 32% of the 5′SSs have an AG//GU without //GURAG (Supplementary Figure S8G), and 16% of the 5′SSs have a +4U (Supplementary Figure S8H) which also strongly favour AG//GU. Our RNA-Seq analysis of *mett-10* sensitive 5′SSs and the presence of different 5′SS classes could mean not all U6 snRNAs are m6A methylated in cells, which could also be a regulated process or cells could use +4A and non-+4A 5′SSs to regulate the expression level of transcript isoforms. Alternatively, regulating spliceosomal proteins that chaperone U6 ACAGA box interactions, such as SNRNP27K, may influence splice site choice.

### The function of METT-10 in mRNA modification is unclear

It was previously reported that m6A methylation of *sams* gene pre-mRNAs by METT-10 is involved in their alternative splicing (35,41). *sams-3,-4 and -5* all have 5′SSs with weak U5 snRNA interacting sequences and a //GURAG motif, which could make these sequences sensitive to the loss of *mett-10*. This could shift the usage of weak upstream 3′SSs to the stronger downstream 3′SSs, as seen for most alternative 3′SS usage events. However, the alternative 3′SSs of *sams-3, -4 and -5* used with low frequency in wild-type animals have a strong UUUCAG/R motif (Supplementary Figure S4). Therefore, METT-10 mediated m6A methylation at the pre-mRNA introns of *sams-3, -4* and *-5* could be a unique event in *sams* genes.

There are biochemical data supporting *in vitro* m6A methylation of *sams* pre-mRNAs by METT-10 (35,41,111), but evidence for *in vivo sams* pre-mRNA m6A methylation is limited. Using the highly sensitive SCARLET method (112), Mendel *et al.* could detect significant U6 snRNA m6A methylation in total RNA, but m6A methylation was undetectable on *sams-3* pre-mRNAs (35). Watabe et al. used ONT-DRS with *in vitro* transcribed *sams-3 and -4* transcripts that are either m6A methylated or not and trained machine learning algorithms, which were then used to classify the endogenous *sams-3 and -4* transcripts in the non-sense mediated decay mutants (*smg-2)*. They concluded that most *sams-3/-4* transcripts are m6A methylated (41). Our comparison of the ONT-DRS data between *mett-10^−/−^* and wild-type animals using two transcriptome-wide analysis tools did not reveal any significant sites of m6A modification within *sams* genes. Therefore, m6A modification of *C. elegans* mRNAs requires further investigation.

### The biological significance of the alternative splicing events

The majority of the splicing events, either *cis*- or *trans*-, have the potential to alter the open reading frame and, therefore, could significantly impact protein expression for hundreds of genes. Indeed, most intron retention and 5′SS events generate out-of-frame changes. Although the majority of the alternative 5′SS events we detected in *mett-10^−/−^* mutants generate out-of-frame changes (55.4%), a significant portion remains in-frame (44.6%). On the other hand, most of the alternative 3′SS events are in-frame, supporting the observation that weak and less frequently used 3′SSs move to strong canonical positions in the absence of U6 snRNA m6A methylation.

Most of the retained outron sequences in *mett-10^−/−^* animals have alternative start codons. Although we do not know if the translation machinery utilises these upstream start codons, genes sensitive to outron retention are more likely to have in-frame start codons within the outron sequences. Our analysis of Kozak sequences around upstream start codons suggests that upstream start codons within outron-retained regions are indistinguishable from the canonical start codons of the outron-retaining genes. In *C. elegans*, SL trans-splicing is more important for translation efficiency compared to the presence of a Kozak sequence (113,114). Therefore, outron retention could generate novel protein isoforms. However, upstream start codons can also interfere with the translation efficiency of canonical start codons and reduce protein expression (113).

In summary, our results show that altering U6 snRNA function through its m6A modification or through proteins that interact with U6 during spliceosome assembly leads to multiple alternative splicing changes.

## Supplementary Material

gkae447_Supplemental_Files

## Data Availability

All raw data related to RNA sequencing is deposited at the European Nucleotide Archive (https://www.ebi.ac.uk/ena/browser/home) under the accession number PRJEB65287. All software tools and adjustments to code have been described in materials and methods.
