# Supplementary material for: U6 snRNA m6A modification is required for accurate and efficient splicing of C. elegans and human pre-mRNAs
Source: Nucleic Acids Res. 2024 May 29;52(15):9139–60. doi: 10.1093/nar/gkae447 (PMC11347140; doi:10.1093/nar/gkae447)
Supplement: gkae447_Supplemental_Files [file gkae447_supplemental_files.zip › Supplemental_figures_Shen_et_al.pdf]

Figure S1

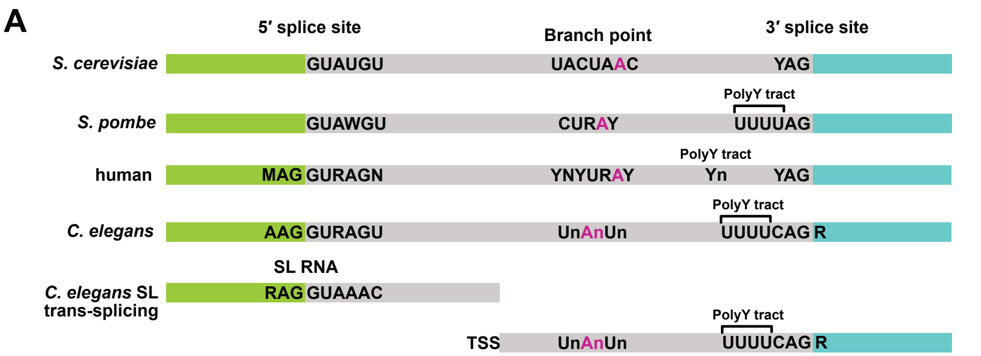

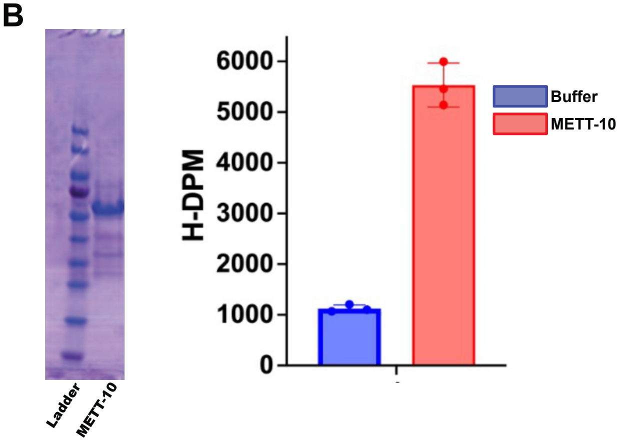

>METT-10 codon optimised_full_length_sequence

ATGAGCCAGAACAATGAAATGCATCCGCGTAATCCGTATCGTAATAAACCGCCTGATTTTAAA
GCACTGGCAGTTGAATATCCGGAATTTCGTAAATTTTGCCAGTATGTGAGCAATGGCAAAGT
GACCTTCGATTTCAAAAAAGATGCAGCAGTTCGTTGTCTGACCCAGACACTGCTGAAAAAA
GATTTTAATCTGGACGTGGAAATTCCGCCTGGTCATCTGGTTCCGCGTGTTCCGCAGAAAC
TGAATTATTGTCTGCTGATTGATGATCTGCTGAAAGCCAATAAACTGACCAAAAACGTGATTG
GCATTGATATTGGCACCGGCACCAGCTGTATTCATGCACTGATTGGTGCACGCCAGTTTAAC
TGGAAATTTATCGCAACCGATGGTGATGAAAAAAGCGTTCGTGTTGCACATGAAAATGTGG
CAAAAAATGGTCTGAGCAGCAGCATTTGTGTTGTTCATGTTAATCCGGATGTTAAGACCGTT
CTGATGGATGTTGTTAATACCATTCCGGACACCGATTATGCCTTTTGTATGTGTAATCCGCCT
TTTTTCGAGAAAGGTAACGGTGATGATAAATTCTGCGAAGATATTAGCAGCTCCACCGAAAC
CTATAGCAATCGTGTTGCCAGCGAATTTCGCACCGCACCGCATAGCGCAACCTTTGCAAGC
AGCGCAGAACTGTTTGTTGATGGTGGTGAAGTTGCATTTGTGAACCGCATTATTGATGATAG
CGTTCTGCTGCGTGATCGCATCAAAATCTATACCACCATGATTGGTCGTAAAAGCAGCCTGA
AACCGCTGCAGAATCGTCTGCAGCGTTTTGGTGATGATGTGAAAATTATGATTAGCGTGCTG
AATCAGGGTAAAACCAAACGTTGGATGCTGGCATGGACCTTTAGCAAAAGCGTTAGCCTGA
CCACCATTGATCGTCCGATTAGCTTTCAGTGTCCGAAACCGGGTCTGACCCGTCTGATGCA
AGAAATTAGCATTCTGAATGGTCGTCTGCGTCAAGAAGATACCCTGGCAATTGTTGCAGAAT
TCAAATGTGTTACCTGGACCAATCAGCGTGCACGTAAACGTGCAAAAGCAATTCTGAGCGA
AAGCAGCATCAAAAAAGCCAAATGGAATTTTAGCAATGTGGCATGTCAGGTTGCATTCGGA
GCCGGTGATGGTAAAGATAGCTATACCGATGCAGGTAATTTTGTTAGCAGCGAAAGTATTCC
GACCAATAATCTGAATGCATGGGATAATGCAAGCCAGGCATATTTTCCGCTGCCGAATGGCG
AAGTTCCGGGTCCGATTATTCGTATTCGCATTCAGGTTTTTAGCGAGGATAGCTATGATAGCA
TCTCCTTTGAACTGATTAGCGGTAGCAAACAGCATCTGCATCAGCTGGTTCAGTATCTGAAA
AATCTGATTTGCCGCTAA

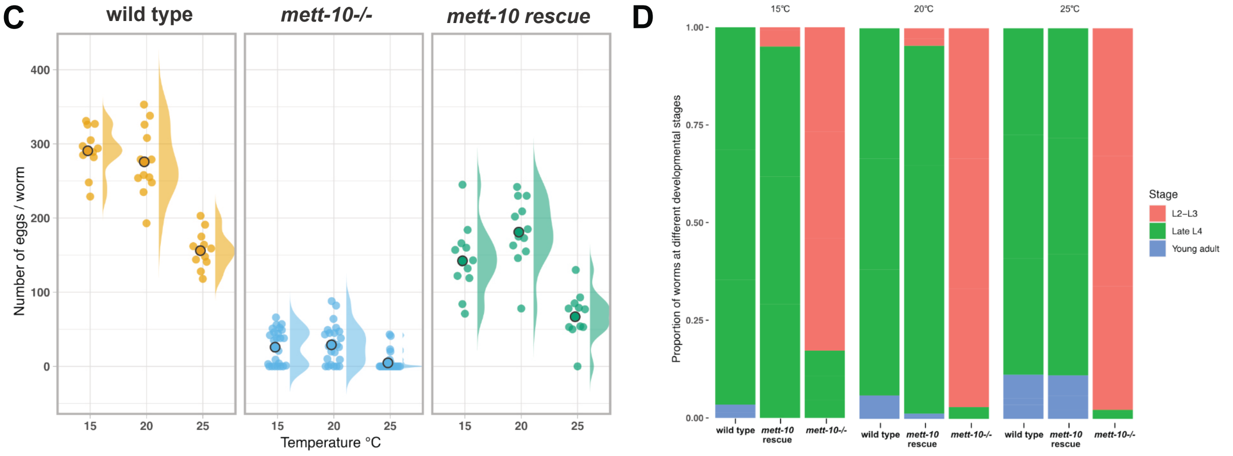

Figure S2

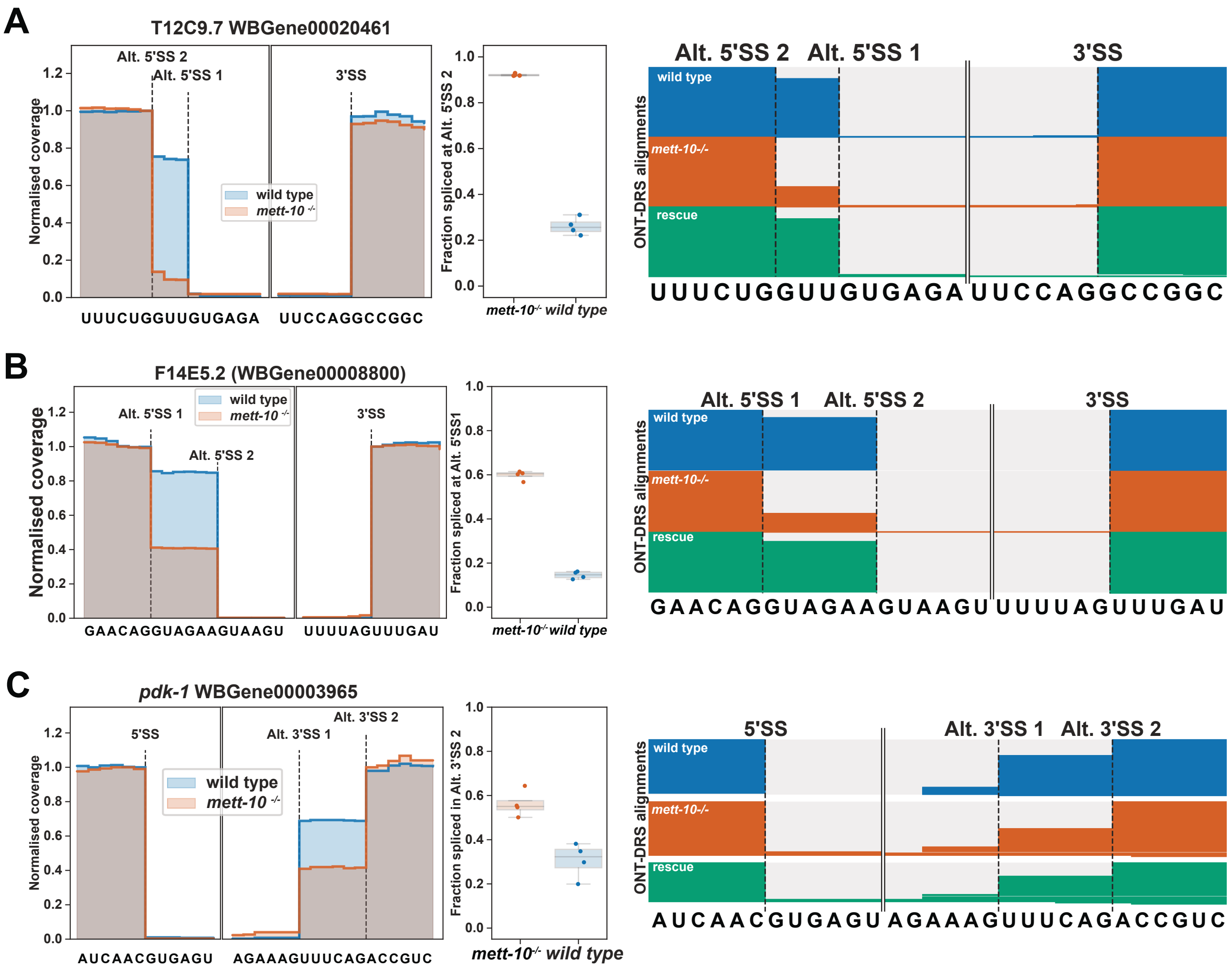

A
T12C9.7 WBGene00020461
Alt. 5'SS 2
Alt. 5'SS 1
3'SS
Normalised coverage
wild type
mett-10 -/-
UUUCUGGUUGUGAGA
UUCCAGGCCGGC
Fraction spliced at Alt. 5'SS 2
mett-10-/-
wild type
Alt. 5'SS 2
Alt. 5'SS 1
3'SS
ONT-DRS alignments
wild type
mett-10-/-
rescue
UUUCUGGUUGUGAGA UUCCAGGCCGGC
B
F14E5.2 (WBGene00008800)
Alt. 5'SS 1
Alt. 5'SS 2
3'SS
Normalised coverage
wild type
mett-10 -/-
GAACAGGUAGAAGUAAGU
UUUUAGUUUGAU
Fraction spliced at Alt. 5'SS1
mett-10-/-
wild type
Alt. 5'SS 1
Alt. 5'SS 2
3'SS
ONT-DRS alignments
wild type
mett-10-/-
rescue
GAACAGGUAGAAGUAAGU UUUUAGUUUGAU
C
pdk-1 WBGene00003965
5'SS
Alt. 3'SS 1
Alt. 3'SS 2
Normalised coverage
wild type
mett-10 -/-
AUCAACGUGAGU
AGAAAGUUUCAGACCGUC
Fraction spliced in Alt. 3'SS 2
mett-10-/-
wild type
5'SS
Alt. 3'SS 1
Alt. 3'SS 2
ONT-DRS alignments
wild type
mett-10-/-
rescue
AUCAACGUGAGU AGAAAGUUUCAGACCGUC

Figure S3

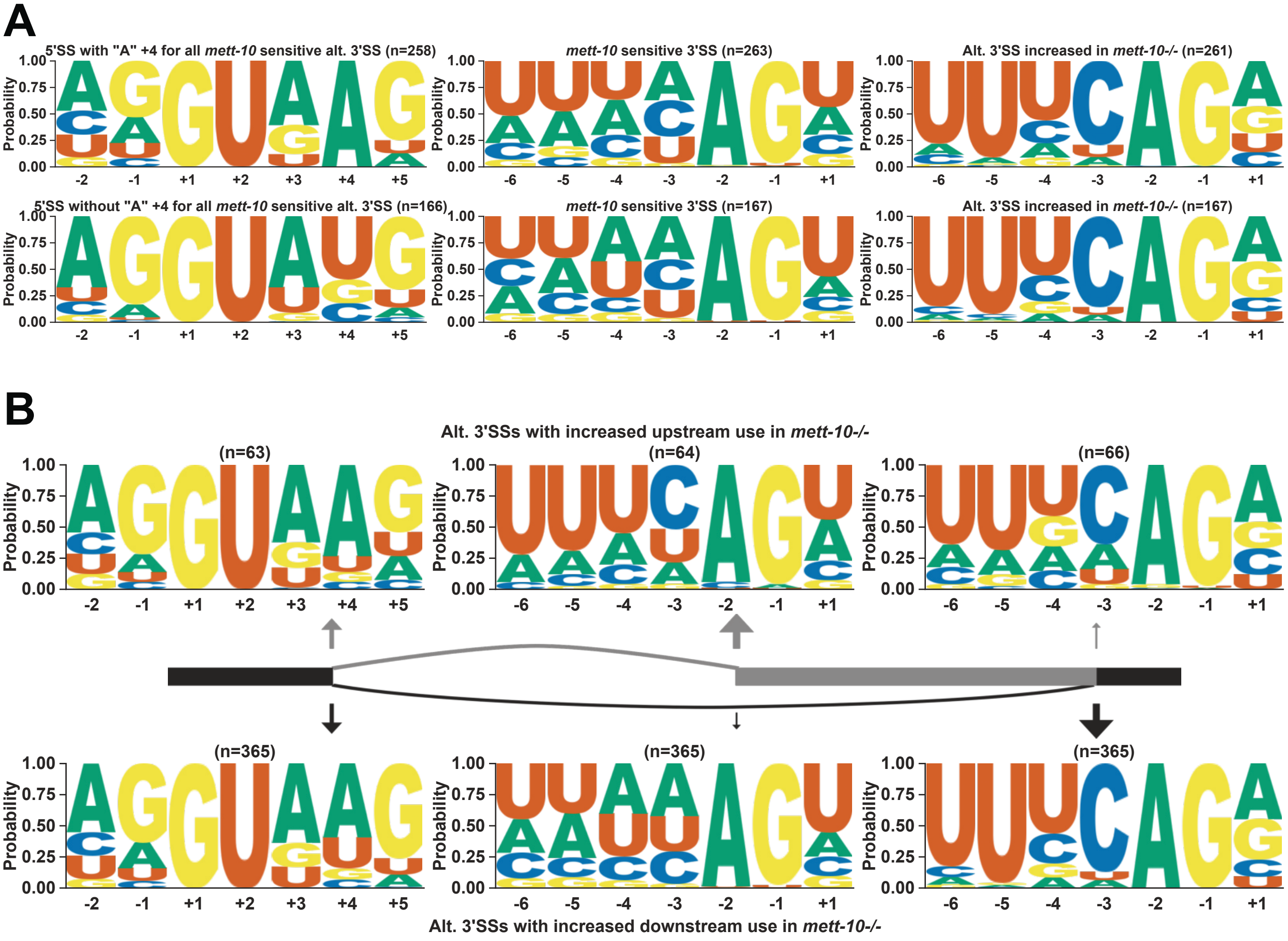
A
5'SS with "A" +4 for all mett-10 sensitive alt. 3'SS (n=258)
mett-10 sensitive 3'SS (n=263)
Alt. 3'SS increased in mett-10-/- (n=261)
5'SS without "A" +4 for all mett-10 sensitive alt. 3'SS (n=166)
mett-10 sensitive 3'SS (n=167)
Alt. 3'SS increased in mett-10-/- (n=167)
Probability
B
Alt. 3'SSs with increased upstream use in mett-10-/-
(n=63)
(n=64)
(n=66)
(n=365)
(n=365)
(n=365)
Alt. 3'SSs with increased downstream use in mett-10-/-

Figure S4

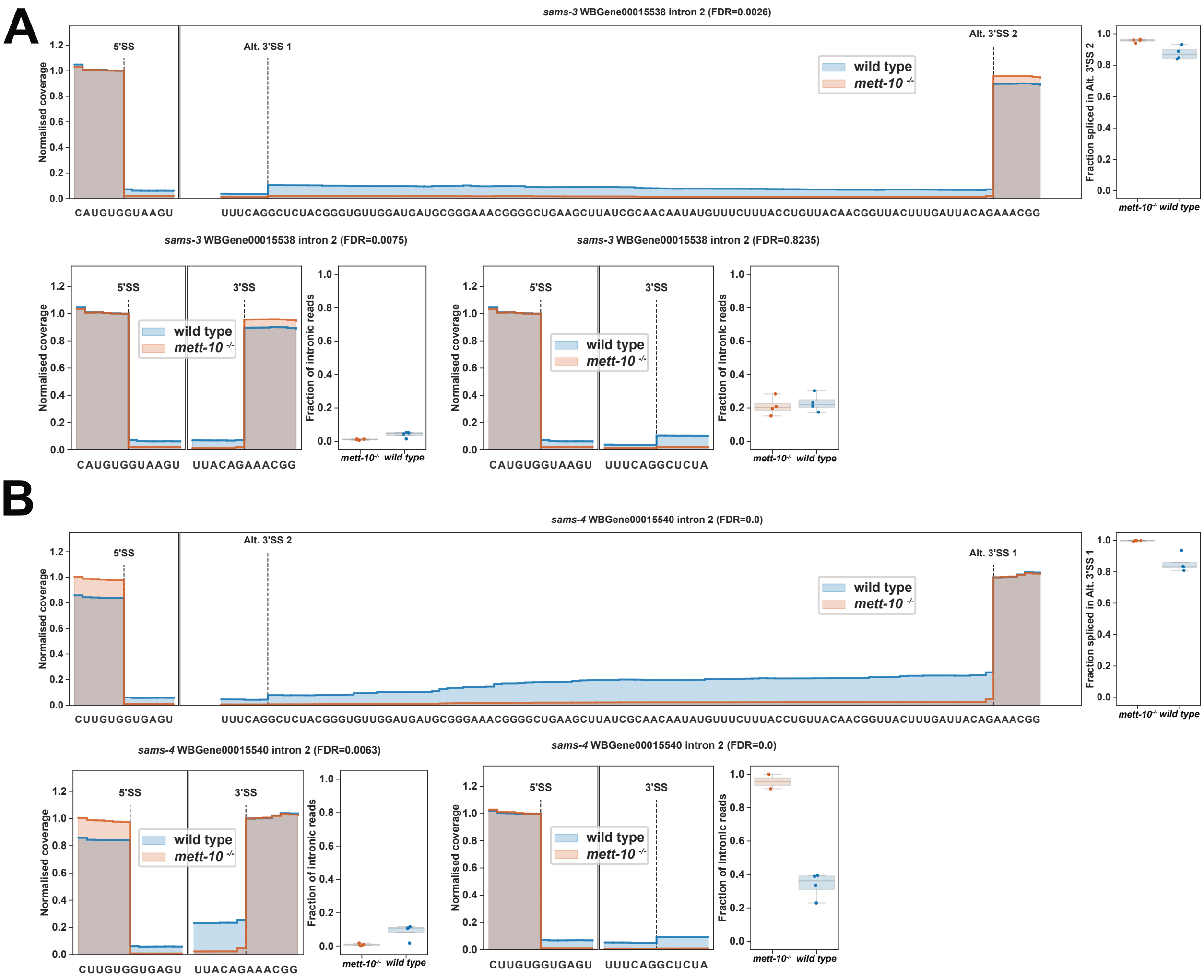
A
sams-3 WBGene00015538 intron 2 (FDR=0.0026)
5'SS
Alt. 3'SS 1
Alt. 3'SS 2
wild type
mett-10 -/-
Normalised coverage
CAUGUGGUAAGU
UUUCAGGCUCUACGGGUGUUGGAUGAUGCGGGAAACGGGGCUGAAGCUUAUCGCAACAAUAUGUUUCUUUACCUGUUACAACGGUUACUUUGAUUACAGAAACGG
Fraction spliced in Alt. 3'SS 2
mett-10-/- wild type
sams-3 WBGene00015538 intron 2 (FDR=0.0075)
3'SS
UUACAGAAACGG
Fraction of intronic reads
sams-3 WBGene00015538 intron 2 (FDR=0.8235)
UUUCAGGCUCUA
B
sams-4 WBGene00015540 intron 2 (FDR=0.0)
CUUGUGGUGAGU
Fraction spliced in Alt. 3'SS 1
sams-4 WBGene00015540 intron 2 (FDR=0.0063)
sams-4 WBGene00015540 intron 2 (FDR=0.0)

Figure S5

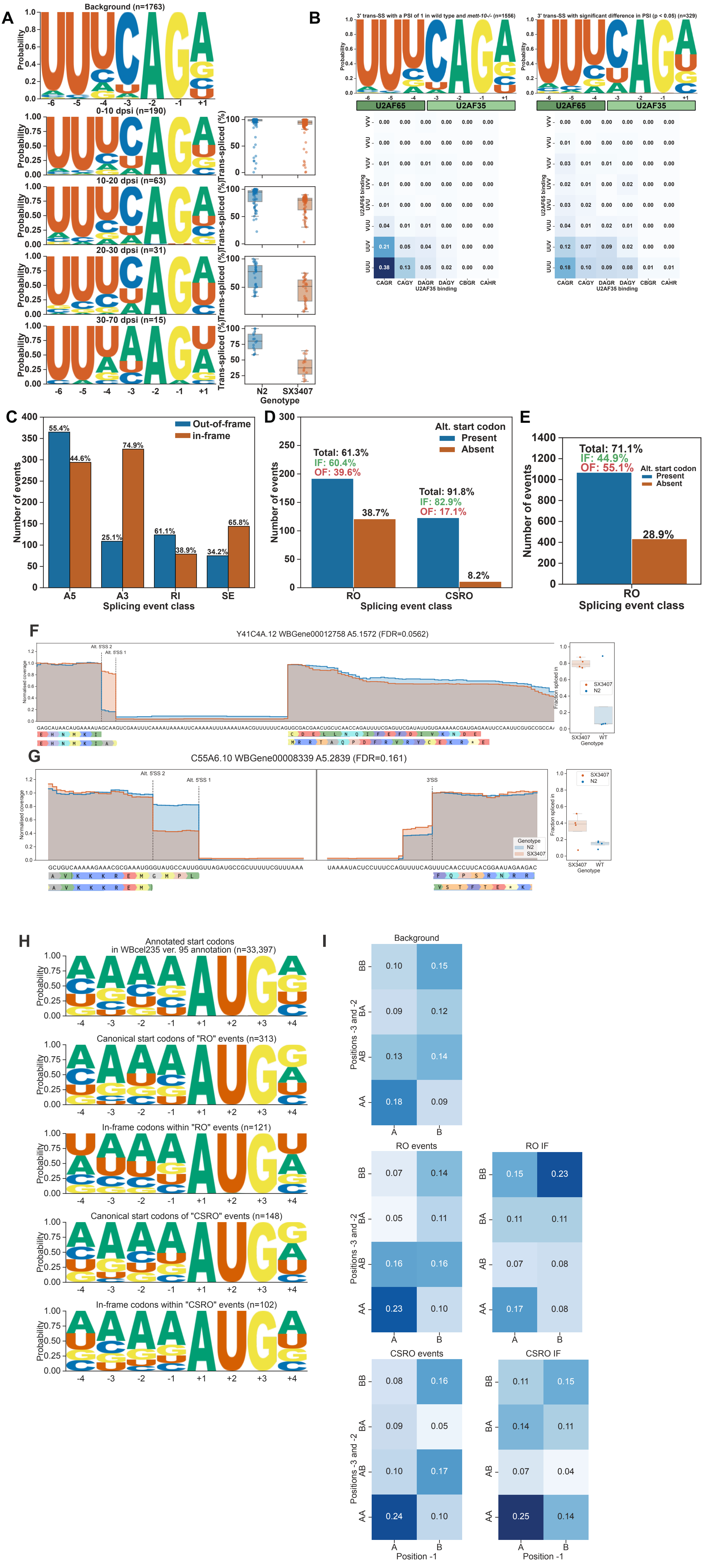
A
Background (n=1763)
0-10 dpsi (n=190)
10-20 dpsi (n=63)
20-30 dpsi (n=31)
30-70 dpsi (n=15)
Probability
Trans-spliced (%)
N2
SX3407
Genotype
B
3' trans-SS with a PSI of 1 in wild type and mett-10-/- (n=1556)
3' trans-SS with significant difference in PSI (p < 0.05) (n=329)
U2AF65
U2AF35
U2AF65 binding
U2AF35 binding
CAGR
CAGY
DAGR
DAGY
CBGR
CAHR
C
Out-of-frame
in-frame
Number of events
Splicing event class
A5
A3
RI
SE
55.4%
44.6%
25.1%
74.9%
61.1%
38.9%
34.2%
65.8%
D
Alt. start codon
Present
Absent
Total: 61.3%
IF: 60.4%
OF: 39.6%
38.7%
Total: 91.8%
IF: 82.9%
OF: 17.1%
8.2%
RO
CSRO
E
Total: 71.1%
IF: 44.9%
OF: 55.1%
28.9%
F
Y41C4A.12 WBGene00012758 A5.1572 (FDR=0.0562)
Alt. 5'SS 2
Alt. 5'SS 1
Normalised coverage
Fraction spliced in
SX3407
N2
WT
Genotype
G
C55A6.10 WBGene00008339 A5.2839 (FDR=0.161)
3'SS
H
Annotated start codons in WBcel235 ver. 95 annotation (n=33,397)
Canonical start codons of "RO" events (n=313)
In-frame codons within "RO" events (n=121)
Canonical start codons of "CSRO" events (n=148)
In-frame codons within "CSRO" events (n=102)
I
Background
RO events
RO IF
CSRO events
CSRO IF
Positions -3 and -2
Position -1

Figure S6

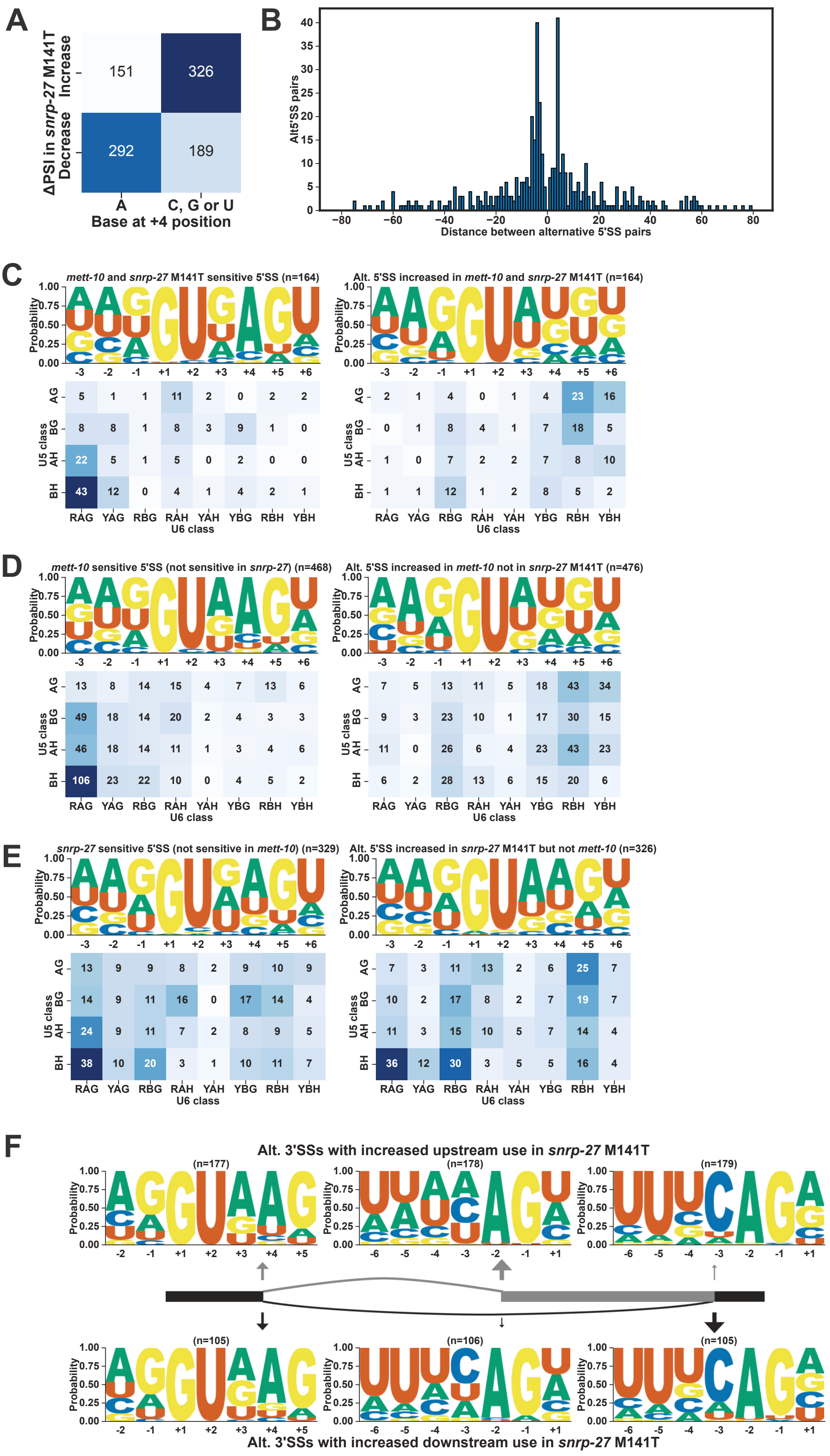
A
ΔPSI in snrp-27 M141T
Decrease
Increase
151
326
292
189
A
C, G or U
Base at +4 position
B
Alt5'SS pairs
Distance between alternative 5'SS pairs
C
mett-10 and snrp-27 M141T sensitive 5'SS (n=164)
Alt. 5'SS increased in mett-10 and snrp-27 M141T (n=164)
Probability
U5 class
U6 class
RAG YAG RBG RAH YAH YBG RBH YBH
D
mett-10 sensitive 5'SS (not sensitive in snrp-27) (n=468)
Alt. 5'SS increased in mett-10 not in snrp-27 M141T (n=476)
E
snrp-27 sensitive 5'SS (not sensitive in mett-10) (n=329)
Alt. 5'SS increased in snrp-27 M141T but not mett-10 (n=326)
F
Alt. 3'SSs with increased upstream use in snrp-27 M141T
(n=177)
(n=178)
(n=179)
(n=105)
(n=106)
(n=105)
Alt. 3'SSs with increased downstream use in snrp-27 M141T

Figure S7

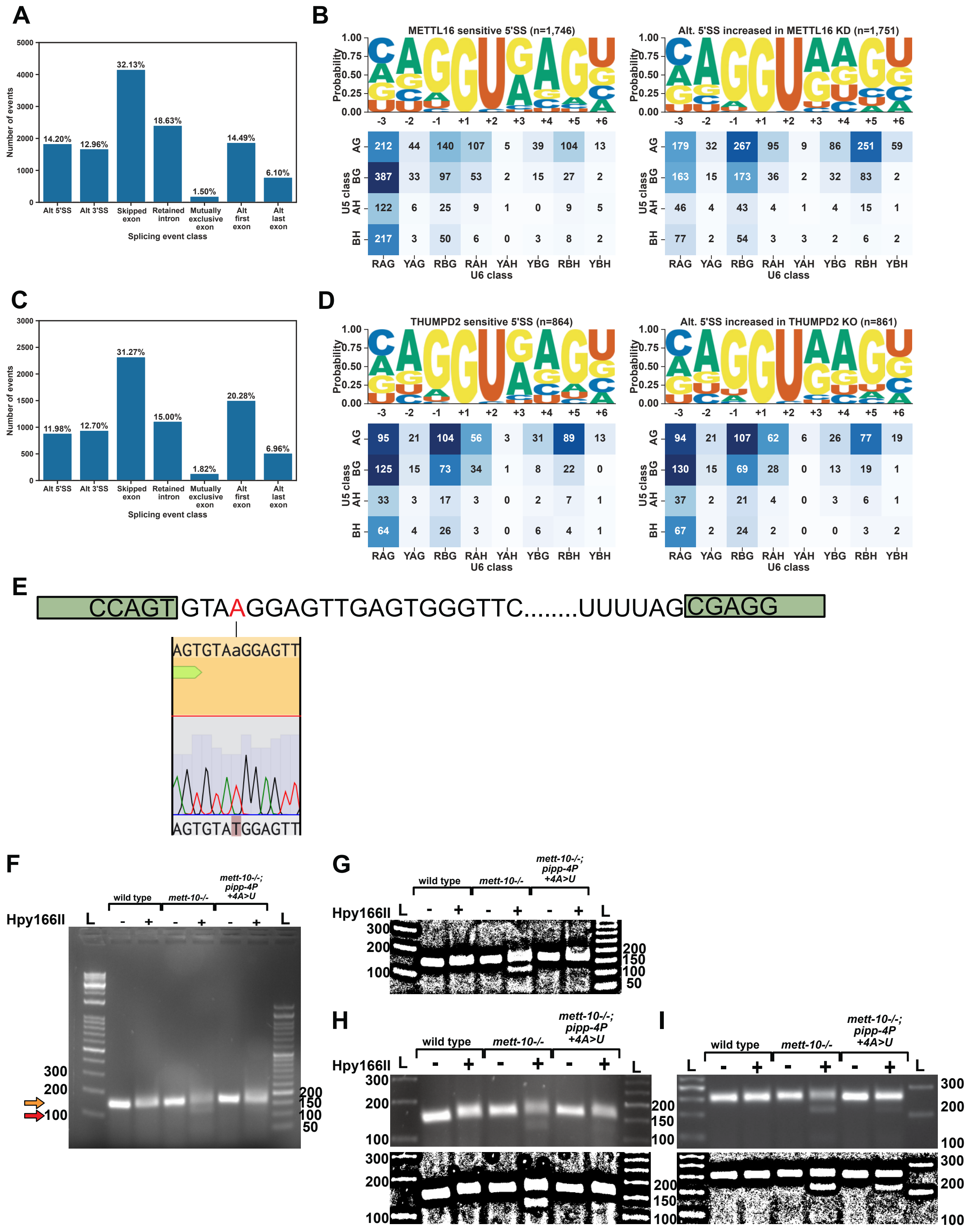
A
Number of events
Splicing event class
14.20%
12.96%
32.13%
18.63%
1.50%
14.49%
6.10%
Alt 5'SS
Alt 3'SS
Skipped exon
Retained intron
Mutually exclusive exon
Alt first exon
Alt last exon
B
METTL16 sensitive 5'SS (n=1,746)
Alt. 5'SS increased in METTL16 KD (n=1,751)
Probability
U5 class
U6 class
RAG YAG RBG RAH YAH YBG RBH YBH
AG BG AH BH
C
11.98%
12.70%
31.27%
15.00%
1.82%
20.28%
6.96%
D
THUMPD2 sensitive 5'SS (n=864)
Alt. 5'SS increased in THUMPD2 KO (n=861)
E
CCAGT GTAAGGAGTTGAGTGGGTTC........UUUUAG CGAGG
AGTGTAaGGAGTT
AGTGTATGGAGTT
F
G
H
I
wild type
mett-10-/-
mett-10-/-; pipp-4P +4A>U
Hpy166II
L
- + - + - +

Figure S8

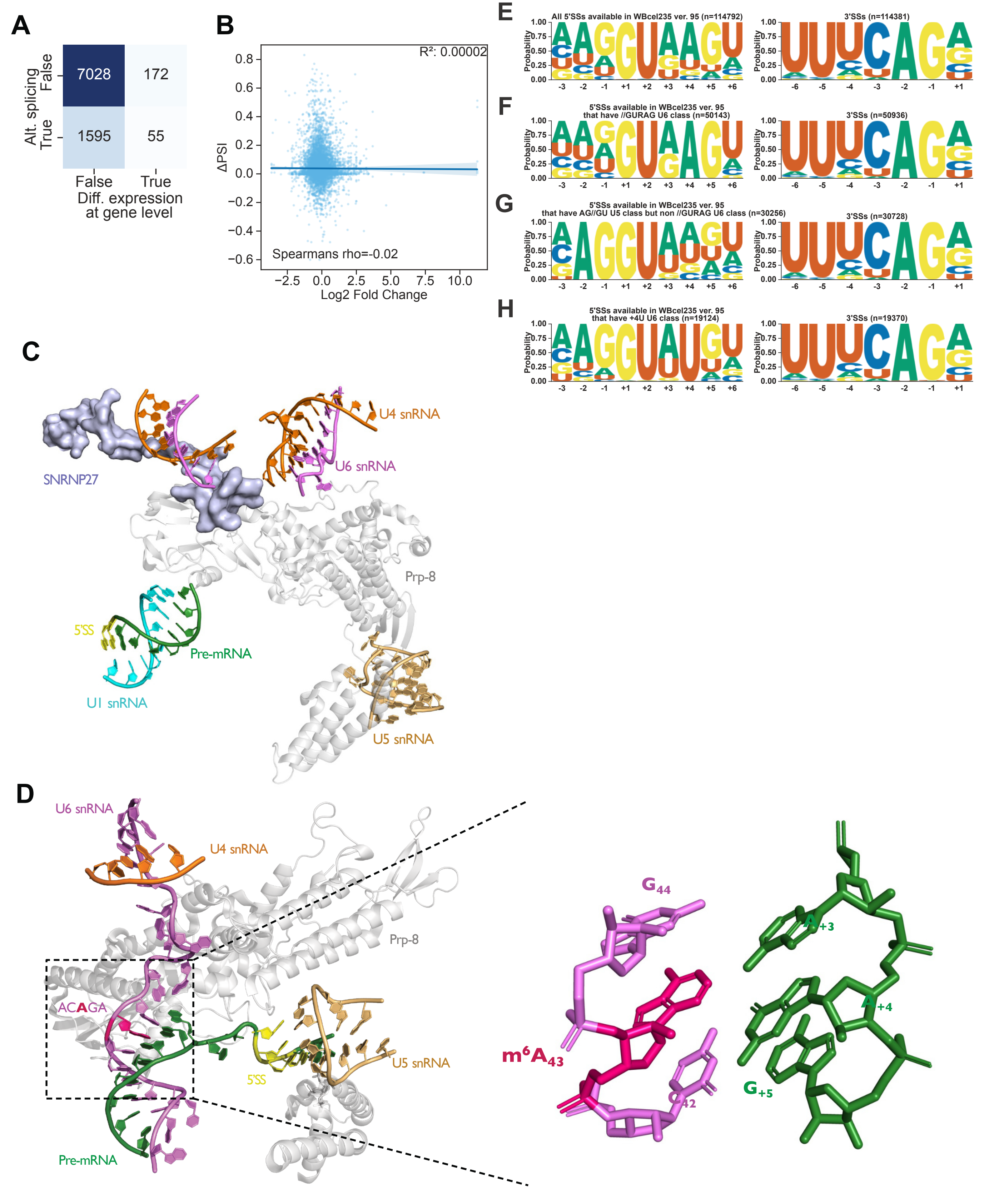
A
Alt. splicing
False
True
7028
172
1595
55
False
True
Diff. expression at gene level
B
R²: 0.00002
ΔPSI
Spearmans rho=-0.02
Log2 Fold Change
C
U4 snRNA
U6 snRNA
SNRNP27
Prp-8
5'SS
Pre-mRNA
U1 snRNA
U5 snRNA
D
U6 snRNA
U4 snRNA
Prp-8
ACAGA
U5 snRNA
5'SS
Pre-mRNA
G44
A+3
A+4
m6A43
G+5
E
All 5'SSs available in WBcel235 ver. 95 (n=114792)
3'SSs (n=114381)
Probability
F
5'SSs available in WBcel235 ver. 95 that have //GURAG U6 class (n=50143)
3'SSs (n=50936)
G
5'SSs available in WBcel235 ver. 95 that have AG//GU U5 class but non //GURAG U6 class (n=30256)
3'SSs (n=30728)
H
5'SSs available in WBcel235 ver. 95 that have +4U U6 class (n=19124)
3'SSs (n=19370)

Figure S1.

Splice site motifs in different organisms, methylation of U6 by METT-10 and *mett-10* phenotypes. **(A)** 5′SS, 3′SS and branch point sequences in *S. cerevisiae, S. pombe, humans, C. elegans and C. elegans* trans-splice sites. 5′SS exons are shown in green, 3′SS exons in turquoise and introns in grey. Y: pyrimidines. TSS: transcription start site. **(B)** Gel electrophoresis of recombinant purified METT-10 (left panel), used in *in vitro* methylation reaction of U6 snRNA (right panel, H-DPM ([3H] disintegrations per minute). *In vitro* methylation was carried out by incubating purified recombinant full-length METT-10 with [3H]-SAM and full-length *in vitro* transcribed *C. elegans* U6 snRNA. Methylated RNA was blotted onto a membrane, and the amount of tritium incorporation was measured using a scintillation counter and shown as disintegrations per minute (H-DPM). Detailed methods are included in the Methods section. Bars correspond to the amount of tritium incorporated into methylated U6 substrate shown as disintegrations per minute (H-DPM). Data are shown as mean ± standard deviation from three replicates. **(C)** Animals expressing the *mett-10 rescue* transgene in the germline and in a *mett-10-/-* background have higher progeny per animal at all 3 temperatures tested. **(D)** Animals expressing the *mett-10 rescue* transgene in the germline and in a *mett-10-/-* background have developmental timing similar to wild-type animals.

Figure S2.

Additional examples of alternative 5′SS and 3′SS usage events. **(A)** T12C9.7 encodes the mitotic specific cyclin B2 and, in wild-type animals, more frequently spliced at the Alt. 5′SS 1 //GUGAG. In *mett-10*$^{-/-}$, most of the splicing shifts to Alt. 5′SS 2 UG//GUUGU. Illumina short-read coverage is on the left, and nanopore direct RNA reads on the right. Analysis of Nanopore reads includes the animals expressing transgenic *mett-10* in the germline in a *mett-10* mutant background. **(B)** F14E5.2 encodes the *C. elegans* orthologue of the human GLG1, and F14E5.2 exon 8 is most frequently spliced at Alt. 5′SS 2 AA//GUAAG in wild-type animals. In *mett-10*$^{-/-}$ animals, 5′SS choice moves to the nearby Alt. 5′SS 1 AG//GUAGA. **(C)** *pdk-1* is the *C. elegans* orthologue of the human cancer-associated kinase PDPK1. *Pdk-1* is frequently spliced in wild-type animals at both Alt. 3′SS 1 and Alt. 3′SS 2. In *mett-10*$^{-/-}$ animals, most splicing events move to the Alt. 3′SS 2.

Figure S3.

Alternative 3′SS usage. **(A)** Most alternative 3′SS events are associated with 5′SSs with +4A (263) as opposed to 5′SSs without +4A, which are enriched for AG//GU (167). *mett-10*$^{-/-}$ sensitive 3′SSs have weak 3′SS motif of UUUCAG/R and there is a switch from these 3′SSs to a strong 3′SS with a clear UUUCAG/R motif in *mett-10*$^{-/-}$. **(B)** Majority of *mett-10*$^{-/-}$ sensitive 3′SS events (365) shift downstream to a stronger 3′SS motif (bottom panel). Alternative 3′SS events that shift upstream are limited (64) and tend to shift to a weaker 3′SS motif, although the difference is less clear.

Figure S4.

sams gene alternative splicing events. **(A)** RNA-Seq coverage of *sams-3* gene alternative splicing events for alternative 3′SS usage and two different intron retention events. **(B)** RNA-Seq coverage of *sams-4* gene alternative splicing events for alternative 3′SS usage and two different intron retention events.

Figure S5.

mett-10 sensitive 3′ trans-splice sites have weak U2AF binding motifs. **(A)** Analysis of 3′ trans-splice site motifs among transcripts with different levels of trans-splicing defects as in Figure 5E. **(B)** Frequency of sequences corresponding to U2AF65 binding (y-axis) and U2AF35 binding (x-axis) alongside the sequence logo of transcripts that do not show trans-splicing defect (left panel) and transcripts that show significant trans-splicing defect (right panel). **(C)** Bar plots showing the percentage of out-of-frame and in-frame changes by each class of cis-splicing events observed in *mett-10* mutant animals. **(D)** Bar plots showing the presence or absence of alternative start codon within the outron retained regions observed in *mett-10* mutant animals. The percentage of in-frame and out-of-frame start codons are shown above the blue bars. **(E)** Presence or absence of alternative start codons within a 120nt window upstream of annotated canonical start codons across *C. elegans* genes that show no outron retention. The percentage of in-frame and out-of-frame start codons is shown above the blue bar. **(F-G)** Examples of genes where alternative 5′SS usage generates a frame-shift in the open reading frame of **(F)** Y41C4A.12 and **(G)** C55A6.10. **(H)** Sequence motif analysis of AUG start codons from positions -4 to +4 for all *C. elegans* annotated start codons, canonical start codons of genes showing retained outron events (RO), in-frame start codons of genes showing RO events, canonical start codons of genes showing cis-spliced retained outron (CSRO) events, and in-frame start codons within CSRO events. **(I)** Heatmap analysis showing different combinations of bases at positions -1, -2 and -3 of start codons.

Figure S6.

SNRP-27 is required for effective cis-splicing. **(A)** Heat-map showing presence or absence of +4A for *snrp-27* sensitive 5′SS events **(B)** Histogram of distance between alternative 5′SS pairs and the number of events. **(C, D and E)** 5′SS sequence motif and the frequency U5 and U6 interacting sequences for 5′SSs sensitive to **(C)** both *mett-10* and *snrp-27*, **(D)** only to *mett-10* and **(E)** only to *snrp-27.* **(F)** Sequence motif 3′SSs that are sensitive to *snrp-27* and the alternative 3′SS usage either shifts upstream (upper panel) or downstream (bottom panel).

Figure S7.

METT-10 function is conserved in humans, and editing +4A sites to +4U can rescue splicing. **(A)** Alternative splicing events were observed upon METTL16 knock-down in 293A-TSO cells compared to control cells. **(B)** Sequence motif analysis and frequency of bases in U5 binding positions (-3 and -1) and U6 binding positions (+3 to +6) in 5′SSs that are sensitive to the absence of METTL16 (left) and 5′SSs used more often in the absence of METTL16 (right). **(C)** Alternative splicing events were observed upon THUMPD2 knock-out in HCT116 cells compared to control cells. **(D)** Sequence motif analysis and frequency of bases in U5 binding positions (-3 and -1) and U6 binding positions (+3 to +6) in 5′SSs that are sensitive to the absence of THUMPD2 (left) and 5′SSs used more often in the absence of THUMPD2 (right). **(E)** *pipp-4P* genomic sequence for exon 4 - intron 4 junction. The +4A position that is edited to +4U is shown in red. Sanger sequencing trace confirming the A>T editing is shown below. **(F)** Uncut agarose gel of Figure 7F. **(G)** Automated local contrast adjustment of the agarose gel image shown in Figure 7F shows that no restriction digestion band is visible in *mett-10-/-; pipp-4P +4A>U* strain. **(H)** The agarose gel image of biological replicate 2 of the same figure as shown in Figure 7F. The bottom panel shows the automated local contrast adjustment of the same agarose gel. No clear restriction digestion band is visible in *mett-10-/-; pipp-4P*

+4A>U strain. **(I)** The agarose gel image of biological replicate 3 of the same figure as shown in Figure 7F. The bottom panel shows the automated local contrast adjustment of the same agarose gel. Restriction digestion band is visible in *mett-10-/-; pipp-4P +4A>U* strain, but the level of the restriction digestion product is much less compared to *mett-10-/-*.

Figure S8.

mett-10 sensitive splicing changes do not correlate with the gene expression changes, sequence motifs of diverse splice sites in *C. elegans* and cryo-EM structures of pre-B and B complexes. **(A)** Heat map showing the overlap of genes with significant change in their expression and splicing. Only 55 genes with an FDR < 0.05 have a change in splicing and expression. **(B)** Correlation of splicing changes in *mett-10* mutants animals compared to wild type (**Δ**PSI) and the log2 fold change values. **(C)** cryo-EM structure of the pre-B complex showing SNRNP27K, U6 snRNA, U1 snRNA, U5 snRNA and pre-mRNA PDB: 6QX9. **(D)** cryo-EM structure of the B-complex showing U6 snRNA - pre-mRNA 5′SS interactions PDB: 6AHD. **(E-H)** Sequence motif of 5′SSs and 3′SSs for **(E)** all *C. elegans* genes, **(F)** with a //GURAG motif, **(G)** AG//GU and not //GURAG and **(H)** +4U only.
